# Mitogenome Phylogenetics: The Impact of Using Single Regions and Partitioning Schemes on Topology, Substitution Rate and Divergence Time Estimation

**DOI:** 10.1371/journal.pone.0027138

**Published:** 2011-11-02

**Authors:** Sebastián Duchêne, Frederick I. Archer, Julia Vilstrup, Susana Caballero, Phillip A. Morin

**Affiliations:** 1 Laboratorio de Ecología Molecular de Vertebrados Acuáticos, Universidad de los Andes, Bogotá, Colombia; 2 Protected Resources Division, Southwest Fisheries Science Center, National Marine Fisheries Service, National Oceanic and Atmospheric Administration, La Jolla, California, United States of America; 3 Centre for GeoGenetics, Natural History Museum of Denmark, University of Copenhagen, Copenhagen, Denmark; Barnard College, Columbia University, United States of America

## Abstract

The availability of mitochondrial genome sequences is growing as a result of recent technological advances in molecular biology. In phylogenetic analyses, the complete mitogenome is increasingly becoming the marker of choice, usually providing better phylogenetic resolution and precision relative to traditional markers such as cytochrome b (*CYTB*) and the control region (*CR*). In some cases, the differences in phylogenetic estimates between mitogenomic and single-gene markers have yielded incongruent conclusions. By comparing phylogenetic estimates made from different genes, we identified the most informative mitochondrial regions and evaluated the minimum amount of data necessary to reproduce the same results as the mitogenome. We compared results among individual genes and the mitogenome for recently published complete mitogenome datasets of selected delphinids (Delphinidae) and killer whales (genus *Orcinus*). Using Bayesian phylogenetic methods, we investigated differences in estimation of topologies, divergence dates, and clock-like behavior among genes for both datasets. Although the most informative regions were not the same for each taxonomic group (*COX1*, *CYTB*, *ND3* and *ATP6* for *Orcinus*, and *ND1*, *COX1* and *ND4* for Delphinidae), in both cases they were equivalent to less than a quarter of the complete mitogenome. This suggests that gene information content can vary among groups, but can be adequately represented by a portion of the complete sequence. Although our results indicate that complete mitogenomes provide the highest phylogenetic resolution and most precise date estimates, a minimum amount of data can be selected using our approach when the complete sequence is unavailable. Studies based on single genes can benefit from the addition of a few more mitochondrial markers, producing topologies and date estimates similar to those obtained using the entire mitogenome.

## Introduction

The circular mitochondrial genome is non-recombining, fast evolving and relatively easy to amplify, making it a popular marker for systematics and phylogenetic analyses of taxa ranging from tunicates to woolly mammoths [1], [2]. Owing to the costs involved in sequencing the entire mitogenome and the desire to obtain comparable data among studies, most analyses have focused on sequencing a relatively small portion of the genome. In many taxa, the most common has been the highly variable non-coding control region *(CR)* (mostly for intraspecific studies) [3], [4], [5], followed by the slightly more conserved cytochrome *b (CytB)* [6], [7]. Cytochrome oxidase I (*COXI*) has been proposed as an appropriate region for genetic barcoding of species [8], [9], [10], although its effectiveness in that role has been questioned for cetaceans and other taxa [11]. In some cases, the use of these genes has resulted in trees with low phylogenetic resolution or contradictory topologies among mitochondrial markers [12], [13].

Recent technological advances have made it easier and more affordable to sequence all ∼16,000 base pairs of the mitogenome, increasing its popularity as a phylogenetic marker. This revolution has been especially important for improving phylogenetic resolution in cetacean studies, compared to traditional use of single mitochondrial markers such as *CytB* and *CR* [14], [15], [16], [17], [18].

Although complete mitogenomes often improve phylogenetic estimation, the linkage of mitochondrial regions means that they are expected to share the same phylogeny. However, the use of different regions (including the whole molecule and single genes) can sometimes produce incongruent results [9], [19], [20], [21], [22], leading to uncertainties in the taxonomy, phylogeography, and divergence dates of a number of groups, including cetaceans [17], [18]. Regions responsible for the higher resolution obtained from complete mitogenomes can be identified for particular taxonomic groups by analyzing results from all mitochondrial markers, providing insights into incongruence in results among studies based on single genes.

In this paper, we evaluate the variability and suitability of mitogenome segments at multiple levels of divergence among cetacean taxa. For this purpose, we use recently published cetacean mitogenome datasets: *Orcinus* [17], Delphinidae [18] and Cetacea [14], [15] (for a limited set of analyses only).

Since our approach assesses the performance of individual genes based on how well they match mitogenomic estimates, it is important to note that mitogenomic phylogenetics will not necessarily reflect the true evolutionary history of a species or taxonomic group, but rather that of the mitochondria only. In some cases there will be clear concordance between the mitochondrial and species trees [23], [24], [25], [26], but in the presence of introgression or incomplete lineage sorting, nuclear markers are needed to resolve the species history [27], [28].

These taxonomic groups were chosen because the use of mitogenomes has yielded phylogenetic estimates with substantially greater resolution than single-region markers. Taxonomic resolution of several groups within Delphinidae [18] such as subfamily Globicephalinae and killer whales (*Orcinus*) was made possible through the use of mitogenomes. In the case of relationships within Delphinidae, recent multi-locus nuclear analysis have shown congruence with mitogenomic-based evidence [26]. These results will likely amount to evidence supporting taxonomic revision of *Orcinus* ecotypes (Transients and Antarctic types B and C as full species, and North Pacific Residents and Offshores as subspecies [17]) and species relationships within Globicephalinae.

Using these datasets we focus on phylogenetic resolution at the family (Delphinidae) and genus (*Orcinus*) taxonomic levels, encompassing an evolutionary timeframe between 11 and 0.7 million years before present (MYBP), where the most remarkable improvements have been observed. The following three questions are addressed: (i) How well do individual genes support topologies generated by the entire mitogenome? (ii) Do the same genes provide levels of support similar to each other and to the entire mitogenome at various taxonomic levels? and (iii) How similar are divergence times estimated with individual genes compared to those from the entire mitogenome?

## Methods

### Datasets and sequence alignments

This study made use of complete mitogenomes for Cetacea, *Orcinus*, and Delphinidae, resulting in three datasets. All sequences were downloaded from GenBank (accession numbers shown in Table S1.).

The Cetacea dataset contains 33 unique sequences, including a broad array of Mysticeti and Odontoceti, as used by Morin et al. [17] to estimate the timing of the radiation of *Orcinus*. This dataset was initially analyzed using calibration priors for nine nodes to estimate divergence times in the cetacean phylogeny (calibrations shown in Table S2). Given that these sequences encompass a wide timeframe of approximately 38 MYBP [14], they provide an ideal dataset for testing fossil calibrations in the context of relaxed-clock models, and for investigating the saturation patterns of frequently used mitochondrial markers such as the *CR*.

**Figure 1:**
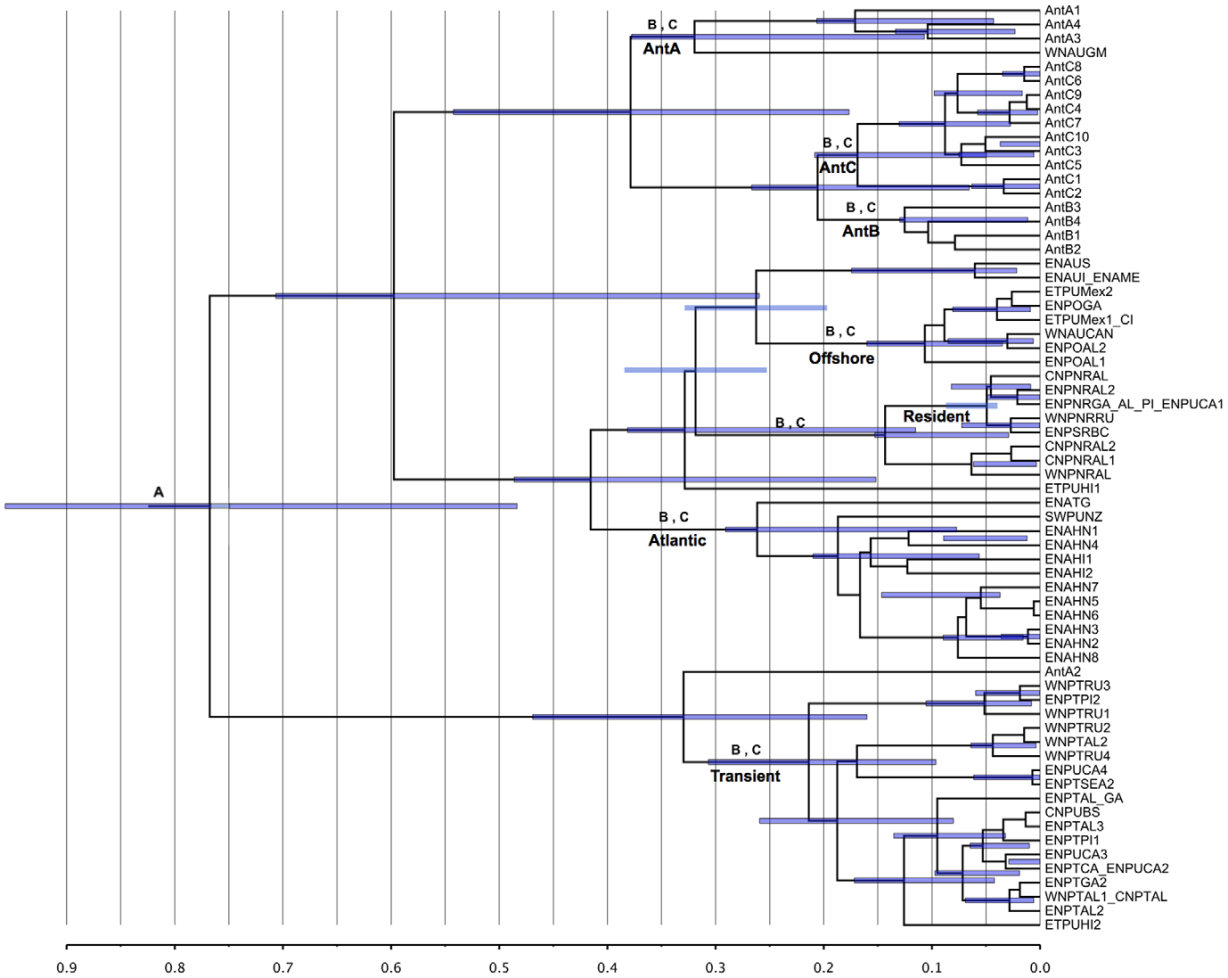
Chronogram for *Orcinus* haplotypes reconstructed using the complete mitogenome. Node labels correspond to: (A) Calibrated nodes, (B) Nodes tested for TMRCA deviation, (C) Nodes tested for PP support. Branch labels correspond to *Orcinus* ecotypes. Antarctic killer whale ecotypes A, B and C are referred to as AntA, AntB and AntC, respectively. Node bars correspond to the 95% HPD for TMRCA of nodes and scale bar represents MYBP (Million years before present).

For *Orcinus* we used a genus-wide phylogeography dataset containing 64 mitogenome sequences from Morin *et al.* [17]. We made use of a single lognormal calibration, corresponding to the origin of all killer whales as shown in Figure 1 and Table S2. Recent molecular evidence suggests that killer whales (currently classified as *Orcinus orca*) may actually contain several subspecies or species [17], [18], [29], [30], but with low genetic diversity and low phylogenetic resolution based on *CR* studies [31], [32], [33]. These putative subspecies/species are currently recognized as different ecotypes or morphotypes according to morphological differences, feeding strategies, and geographic distribution [34], [35], [36], [37]. Complete mitogenomes revealed divergence times between 0.135 and 0.7 MYBP, and high phylogenetic resolution for ecotypes [17], compared to low resolution and recent divergence times (0.03 MYBP) inferred from *CR* sequences [31], [38].

**Figure 2:**
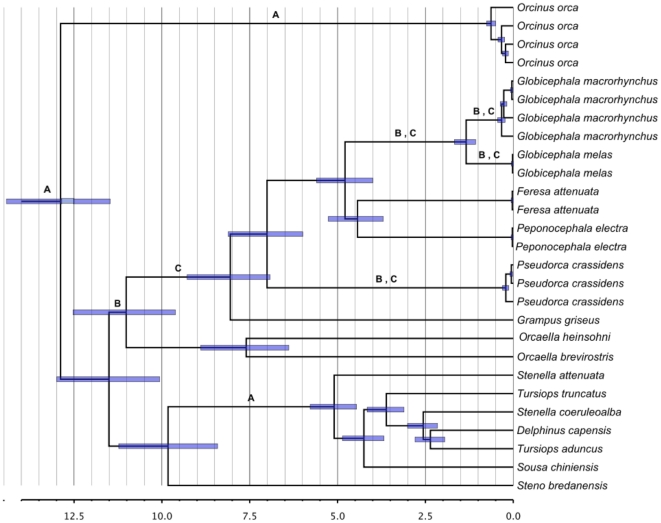
Chronogram for Delphinidae sequences reconstructed using the complete mitogenome. Node labels correspond to: (A) Calibrated nodes, (B) Nodes tested for TMRCA deviation, (C) Nodes tested for PP support. Branch labels correspond to taxonomic groups within Delphinidae. Bars correspond to the 95% HPD for TMRCA of nodes and scale bar represents MYBP.

The Delphinidae dataset is a broad sampling of 31 representatives of 15 species in the family. These data were analyzed using calibrations for three nodes shown in Figure 2 and Table S2. Complete mitogenomes have proved useful in resolving taxonomic uncertainty regarding the placement of several delphinids [18], in contrast to the lower resolution found using *CR*, *CytB*, and nuclear markers [14], [39], [40]. This family has an estimated time to the most recent common ancestor of approximately 11.7 MYBP [14], making this a useful group for studying divergence date estimates. Moreover, the taxonomic uncertainty in subfamilies such as Globicephalinae make it possible to test for monophyly of mitogenome-supported clades.

All mitogenomic datasets listed above were aligned using Clustal W as implemented in Geneious v3.6.1 [41] and manually inspected for reading-frame matching of protein-coding regions.

To evaluate individual gene performance for *Orcinus* and Delphinidae, individual gene sets were extracted to produce 15 separate alignments in addition to the mitogenome: *12S* and *16S* in a concatenated dataset because of their similar evolutionary patterns [42], the thirteen protein-coding genes and the *CR*. Given that genes were analyzed as single entities, the complete individual gene sequences were used, including overlapping sections of between 1 and 16 nucleotides.

### Phylogenetic analyses

Substitution model selection for all individual genes and gene sets was performed using PHYML[43] as implemented in JMODELTEST v1.0 [44]. Best-fitting models according to the Bayesian Information Criterion (BIC) (Table S3) were used for all phylogenetic analyses (sample size of 1000) since this method is known to perform better than other criteria [45]. Saturation plots were compared for the *CR*, the most informative genes (see below), and the complete mitogenome in the cetacean dataset, using standardized JC69 and raw distances. These models were chosen to assess the magnitude of saturation if not accounted for in the model.

Phylogenetic estimates were obtained using BEAST v1.5.4 [46]. Bayesian settings for all alignments of the three sequence datasets were: BIC selected nucleotide substitution model (Table S3) and 3 codon site partitions for protein coding regions assuming relative rates per codon site but not different clocks to avoid overparameterization of short sequences; MCMC chain length of 100 million, sampling every 1000 iterations; Yule speciation process as tree prior since it is more appropriate for haplotypes of different species [47]; Uncorrelated relaxed lognormal molecular clock model, to account for rate variation among lineages and an estimation of how clock-like the data are; Two independent runs to test for chain convergence.

Tracer v1.5.0 [48] was used to check convergence of the Markov chains and to ensure sufficient sampling. In all cases the chains were run long enough to achieve high effective sample sizes (ESS≥1000) for all parameters.

### Analyses of topology and phylogenetic resolution

Topologies estimated from each gene were compared to those estimated from the complete mitogenome for both the Delphinidae and *Orcinus* datasets. This analysis was based on the assumption that the complete mitogenome provided the most reliable results and that individual genes represent imperfect subsamples of the complete molecule.

Posterior probabilities of clades previously identified by Morin et al. [17] for *Orcinus* and Vilstrup et al. [18] for Delphinidae, were used to test for reconstruction of important features of each tree. One maximum-clade-credibility tree, with branch lengths measured in units of time (ultrametric tree), was identified from the trees sampled in the BEAST analyses (removing 10% burn-in), for each gene for both datasets (*Orcinus* and Delphinidae).

After assessing the phylogenetic resolution of individual genes relative to posterior probabilities for clades resolved by the mitogenome, we selected sets of genes that represented the minimum amount of data necessary to reproduce the primary features of the entire mitogenome-based tree in the Delphinidae and *Orcinus* datasets separately. This approach is less time consuming and likely as effective as testing all possible gene combinations. The first step in identifying informative genes was to select those that supported clades that no other individual genes supported (Table 1 and Figures 1 and 2). The second step was to select genes that supported the largest number of clades, even if the clades were supported by more than one gene. Finally, we evaluated whether combining these informative genes only in a single concatenated matrix (or gene subset dataset) would provide support for all clades supported by the mitogenome, therefore producing mitogenome-level support for all clades of interest and a minimum amount of data necessary to reproduce mitogenomic resolution. Informative gene subsets were concatenated and analyzed in BEAST using the same procedure as for individual genes.

**Table 1:** Posterior probability of the highest clade credibility trees.

	*COX1* ^1,2^	*ND4* ^2^	*CYTB* ^1^	*ND1* ^2^	*ND3* ^1^	*CR*	*12S16S*	*ATP8*	*COX3*	*ATP6* ^1^	*ND4L*	*ND2*	*ND5*	*COX2*	*ND1, COX1, ND4*	*COX1, ATP6, ND3, CYTB*	Mitogenome
^(*O*)^ AntA	0.99	0.56	0.99	0.67	0	0.59	0.67	0	0	0	0	0.77	0.06	0	-	1	1
^(*O*)^ AntB	0	0	1	0	0.62	0	0	0	0	0	0	0	0	0	-	1	1
^(*O*)^ AntC*	0	0	0	0	0.94	0	0	0	0	0	0	0	0	0	-	1	1
^(*O*)^ Atlantic	0.11	0	0.99	0.92	0	0	0	0	0	0	0	0	0.08	0	-	1	1
^(*O*)^ Offshore*	0.87	0	0	0	0	0	0	0	0	0	0	0	0.08	0	-	1	1
^(*O*)^ Resident	0	0.99	0	0	0	0	0	0.99	0	0.99	0	0	0	0	-	1	1
^(*O*)^ Transient	0.99	0.93	0.99	0.22	0.53	0.69	0.55	0.94	0.9	0.92	0.92	0.93	0.21	0.68	-	0.99	1
^(*O*)^ AntB+AntC	1	0.96	0	0.98	0.9	1	0.98	0	0.98	0	0.97	0	0.98	0	-	1	1
^(D)^ *Globicephala macrorhynchus*	0.99	1	1	1	0.5	0.99	0.97	0.99	1	0.97	0.99	1	0.99	0.99	1	-	1
^(D)^ *Globicephala melas*	1	1	1	0.8	1	1	0.97	0.98	1	1	0.85	0.99	1	1	1	-	1
^(D)^Globicephalinae	0.97	0.6	0	0.91	0	0	0	0	0	0	0	0	0	0	1		1
^(D)^ *Pseudorca*	1	1	1	1	1	1	0.93	0.99	1	0.99	1	1	0.99	1	1	-	1
^(D)^ Globicephala	1	1	1	1	1	1	0.82	0.99	1	1	0.99	1	0.99	1	0.99	-	1

(D) Taxonomic groups from the Delphinidae dataset.(*O*) Taxonomic groups from the *Orcinus* dataset.1 Most informative genes for *Orcinus.*2 Most informative genes for Delphinidae.* Clades supported by one single gene.

Values correspond to nodes shown in Figures 1 (*Orcinus*) and 2. (Delphinidae). Genes are ordered by the number of groups supported with posterior probabilities above 0.6 across both taxonomic groups.

The similarities between the maximum clade credibility tree topologies produced by individual genes were compared using the APE package in R by estimating the PH85 distance [49], defined as twice the number of different bipartitions between a pair of trees. The resultant pairwise tree distances were then used to create a Neighbor-Joining (NJ) dendrogram of the gene tree distances, representing gene groupings by topology similarities [50], [51].

We then conducted a test based on posterior tree distributions to test whether any mitochondrial partition could represent the mitogenomic topology. We investigated whether the highest clade credibility tree produced by the mitogenome was contained within the 95% credible set of trees of individual genes: First we obtained the trees corresponding to the 95% HPD (Highest Posterior Density) interval of the posterior tree likelihood for every partition and then calculated their PH85 distance to the mitogenomic tree (highest clade credibility tree). If the distance was zero for any of the trees evaluated, then the mitogenome topology was found within the tree set, and we concluded that the particular gene or gene subset could produce a reliable estimate of the mitogenomic tree. This was performed using the APE package in R and customizing functions for our data.

### Gene suitability for date estimation

The ability of different genes to estimate the time to the most recent common ancestor (TMRCA) was assessed on the basis of their coefficient of rate variation estimated in the BEAST analysis for the Delphinidae and *Orcinus* datasets. The coefficient of rate variation is defined as the standard deviation of the rate divided by the mean, with values close to 0 implying a good fit to the strict molecular clock (low rate variation across all lineages) and higher values implying among-lineage rate variation, or deviation from the strict molecular clock [47]. The coefficient of rate variation can substantially exceed 1 if there is extremely low sequence variability and high rate heterogeneity, suggesting that the data are unsuitable for date estimation [52].

Posterior age estimates of nodes within Delphinidae and *Orcinus* were compared for all genes. For this purpose, a one-way Analysis of Variance (ANOVA) was performed on log-transformed TMRCA estimates using the Stats package in R [50].

The posterior age distributions for nodes within Delphinidae and *Orcinus* produced by the mitogenome are referred to as the target (expected) distributions and those of individual genes are the observed distributions. Using the MASS package in R, the expected distributions were log-transformed and fitted to normal distributions, producing mean and s.d. values of a target distribution [53].

The probability of each observation (samples from an observed TMRCA distribution) being over the target distribution was calculated using the pnorm function in the Stats package in R, producing values between 0 (low probability of being above the target distribution) and 1 (high probability of being above the target distribution) for every observation. We then quantified the amount of bias (over or underestimation compared to mitogenomic estimates) using the proportion of observations with probability >0.5 of being over the target distribution per node per partition analysis.

A bias value of 0.5 implies no bias in TMRCA (observations are equally distributed below and above the target), values above 0.5 imply that observations are distributed over the target, suggesting positive bias of TMRCA (overestimation) and values below 0.5 imply negative bias (underestimation). We estimated bias values for all nodes, but in order to address the largest possible deviation from mitogenomic estimates we chose the internal node displaying the largest TMRCA bias for each dataset for subsequent analysis and discussion.

### Accuracy of TMRCA estimates for non-calibrated nodes

Accuracy of divergence time estimation in Bayesian phylogenetic analyses relies on the precision of fossil calibrations and rate constancy among lineages, as well as a range of other factors such as the choice of clock model [54]. We examined the reliability of mitogenome TMRCA estimates by sequentially removing fossil-based priors from nodes being used as calibration points, and comparing their posterior TMRCA (for the nodes with calibration priors removed) with their fossil-based distributions. This cross-validation procedure was conducted only on the cetacean and Delphinidae datasets, with the removal of one calibration per iteration. This method of comparing posteriors with priors is similar to methods proposed by Sanders and Lee [55] to evaluate calibrations.

### Effect of partitioning on phylogenetic estimation

Differences between posterior topology and divergence date estimates of Delphinidae and *Orcinus* were compared among four partitioning schemes of the subsets of the phylogenetically most informative genes (chosen on the basis of their similarity to complete mitogenome results, as described above): Unpartitioned three or four informative genes (1 partition), three codon sites (3 partitions), between genes only (3 or 4 partitions, depending on the sequence dataset) and between genes and codon sites (9 or 12 partitions, depending on the sequence dataset).

Posterior date estimates were compared by performing a one-way ANOVA test of the log-transformed posterior distributions using the Stats package in R, and estimates of topologies were compared to those from the mitogenome using the 95%-credible set of trees as described above for single-gene analyses.

Finally, Bayes factors [56] were used to determine the best partitioning scheme for the informative gene subsets. These tests were performed using the harmonic mean as estimated in Tracer and R (using the Boot package and programming the functions). In both cases 1000 bootstrap replicates were used to obtain standard errors [48], [57], [58].

## Results

### Evolutionary models

Using the BIC, the HKY substitution model was selected for all alignments in *Orcinus* except for *ND3* and *CR*, where HKY+G was preferred. In Delphinidae, a larger range of models was selected; GTR+G for *12S* and *16S*, HKY+I+G for *COX1* and *CR,* and HKY+G for the rest of alignments.

While the HKY and HKY+G models were used for *ND6* (in *Orcinus* and Delphinidae, respectively), estimation of *Kappa* (transition-transversion ratio) resulted in near infinite values and lack of convergence in BEAST. Optimizing the substitution matrix resulted in 5 orders of magnitude more transitions than transversions, therefore explaining the difficulty in estimating *Kappa.* Since models that neglected estimation of *Kappa* were not found within the 95% HPD BIC score, *ND6* was excluded from further phylogenetic analyses.

### Topology

Posterior probabilities (PP) for the eight *Orcinus* and four Delphinidae clades are shown in Table 1 and correspond to the nodes in Figures 1 (*Orcinus*) and 2 (Delphinidae), highest clade credibility trees for all analyses are shown in Figure S1. PP above 0.6 were considered the minimum threshold for support of monophyly for the clades of interest. Based on this criterion, there were clear differences among clades in the number of genes providing support, and between genes and gene subsets in the clades supported. This was especially the case when comparing to full mitogenomic results, which provided high posterior probabilities (PP>0.99) for all groups of interest. This variation in support was higher for *Orcinus* than for Delphinidae, where a majority of clades were supported by most of the genes.

In *Orcinus*, all clades except Transients and Antarctic B and C had low support from most individual genes, whereas in Delphinidae there was overall higher support for clades of interest except when considering the inclusion of *Orcaella* within Globicephalinae (as a basal lineage), as suggested by complete mitogenome analyses [18].

The most informative genes chosen for *Orcinus* were *COX1*, *CYTB*, *ND3* and *ATP6*. *COX1* provided high support for five out of the eight clades, *CYTB* and *ND3* uniquely supported two clades (Atlantic and Antarctic C, respectively), and *ATP6* supported the Residents clade while presenting a lower coefficient of rate variation than other regions supporting this group.

In Delphinidae *ND1*, *COX1* and *ND4* were chosen. These genes highly supported all clades of interest (PP>0.8), except for *ND4,* which only weakly supported the inclusion of *Orcaella* within Globicephalinae (PP = 0.6).

These two gene subsets provided strong support for all clades of interest (Table 1). In two experiments (not shown), removal of any one of the three (Delphinidae) or four (*Orcinus*) genes from the most informative sets resulted in overall lower PP for the Delphinidae and loss of support for an entire clade in *Orcinus*, pointing to these subsets as candidates for the minimum amount of data necessary to obtain mitogenomic resolution.

Removing the informative gene sets from the complete mitogenome demonstrated that overall support was weakened whilst recovering the complete mitogenome topology (not shown), displaying PP values between 0.4 and 0.8 for clades of interest (compared to PP = 1 using the mitogenome). This experiment proved that the remaining mitogenome sequence was not in conflict with the informative genes, however these are necessary for a highly supported topology.

**Figure 3:**
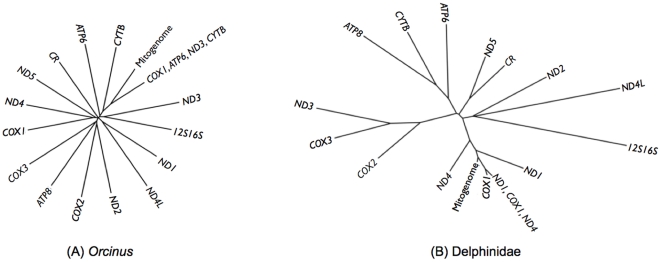
Neighbor-Joining dendrogram for distance between topologies between trees for the *Orcinus* (A) and Delphinidae (B) sequence datasets.

Figure 3 shows the NJ dendrograms for the PH85 topology distance between individual genes, gene subsets and the mitogenome for *Orcinus* (Figure 3A) and Delphinidae (Figure 3B). Branch lengths in the dendrogram for Delphinidae indicate relatively short distances between the informative genes (*COX1*, *ND1*, *ND4*) and mitogenome compared to the rest of the genes, suggesting they are equally different from the rest of the genes. This result demonstrates that beyond support for the clades of interest and their PP, overall topologies for the most informative genes are more similar to that of the mitogenome than any other individual gene analyzed.

The *Orcinus* dendrogram (Figure 3A) is remarkably different from that of the Delphinidae (Figure 3B). There is a star-like pattern in the topology with long terminal branches, suggesting that no two genes produced similar topologies. Furthermore, they are almost equally different among them, pointing to little phylogenetic congruence among single gene analyses. However, the topology for the informative gene subset (*COX1*, *ATP6*, *ND3* and *CYTB*) was the closest to that of the mitogenome, as indicated by the shorter branches and location in the NJ dendrogram. This was consistent with PP results for the clades of interest (Table 1).

Analysis of the 95% credible set of trees from genes and gene subsets in the delphinids showed that the complete-mitogenome tree was contained within the informative subset (*ND1*, *COX1* and *ND4*) but not in any single-gene set of trees, suggesting that only the informative subset of genes could produce a reliable mitogenomic tree. In contrast, in *Orcinus* none of the analyses produced the exact mitogenomic topology in terms of the credible set of trees or the highest clade credibility tree, even though the PH85 distances for the informative gene subset were substantially smaller, as expected from the NJ dendrogram of tree distances.

### Substitution rates

Saturation plots for standardized distances (Figure S2) revealed no observable differences in saturation between the Delphinidae and *Orcinus* datasets. Substantial difference in saturation patterns in different regions of the mitogenome was only observable in the Cetacea sequence set, where the *CR* presented saturation within less sequence divergence than other regions and a remarkably different pattern from that of the mitogenome and the informative gene subsets. In agreement with previous findings [59], this adds to evidence of earlier saturation in the *CR* than in other regions.

Median estimates for individual gene substitution rates are shown in Table 2. In the *Orcinus* data, the fastest median rate was observed for the *CR* and the slowest for *12S* and *16S*. Conversely, the Delphinidae dataset had a relatively homogeneous rate across all genes, with consistent overlap of the 95% HPD.

**Table 2:** Median time to the most recent common ancestor (TMRCA) and bias in date estimation for each gene compared to the mitogenome for Transient killer whales (*Orcinus*) and subfamily Globicephalinae (Delphinidae).

	Transient killer whales			Globicephalinae		
	Median TMRCA	95% HPD	Bias	Median TMRCA	95% HPD	Bias
***12S16S***	0.37	0.13–0.72	0.92	12.13	8.89–14.33	0.88
***ND1***	0.64	0.35–0.94	0.87	8.11	5.96–10.65	0.74
***ND2***	0.36	0.13–0.71	0.92	6.74	4.94–8.88	0.57
***COX1***	0.31	0.13–0.54	0.66	7.36	5.50–9.33	0.6
***COX2***	0.39	0.15–0.73	0.95	9.05	6.11–12.50	0.84
***ATP8***	0.41	0.17–0.76	0.94	6.29	3.54–10.17	0.73
***ATP6***	0.39	0.15–0.73	0.92	10.71	7.18–10.50	0.75
***COX3***	0.41	0.17–0.77	0.97	8.33	5.71–11.33	0.82
***ND3***	0.49	0.20–0.85	0.94	12.2	8.39–14.48	0.95
***ND4L***	0.41	0.16–0.78	0.98	10.63	6.70–13.90	0.9
***ND4***	0.39	0.15–0.73	0.87	7.42	5.74–9.36	0.59
***ND5***	0.4	0.16–0.76	0.75	8.06	6.28–9.90	0.72
***CYTB***	0.32	0.14–0.58	0.78	6.7	4.75–8.74	0.63
***CR***	0.32	0.14–0.55	0.78	8.32	5.83–12.70	0.85
***ND1,COX1,ND4***	-	-	-	7.81	6.48–9.36	0.44
***COX1,ND3,ATP6,CYTB***	0.23	0.10–0.41	0.51	-	-	-
**Mitogenome**	0.2	0.06–0.45	0.5	8.07	6.94–9.25	0.5

Bias values above 0.5 imply overestimation and below 0.5, underestimation. Values shown correspond to nodes in Figures 1 (*Orcinus*) and 2 (Delphinidae).

The mitogenome estimated rate was 2.6×10^−3^ (1.50×10^−3^–3.90×10^−3^ 95%HPD) substitutions/site/MY for killer whales and 4.2×10^−3^ (3.70×10^−3^–4.76×10^−3^ 95%HPD) substitutions/site/MY for delphinids. This is similar to previous estimates in cetaceans at 6.0×10^−3^ (5.48×10^−3^–7.26×10^−3^ 95%HPD) substitutions/site/MY [60], suggesting that variation may be due to differences in sample size or genetic diversity of the taxonomic group. The *CR* rates (4.4×10^−3^ (2.32×10^−3^–7.58×10^−3^ 95%HPD) for *Orcinus* and 6.1×10^−3^ (4.47×10^−3^–7.48×10^−3^ 95%HPD) substitutions/site/MY for Delphinidae also agree with previous reports: 5.2×10^−3^ –10.3×10^−3^ substitutions/site/MY based on divergence between Commerson's dolphin (*Cephalorhynchus commersonii*) and *Orcinus* [61], and 4×10^−3^–18×10^−3^ substitutions/site/MY based on Balaenopteridae [62]. However, it is slower than that estimated using coalescent approaches for a portion of the *CR* (3×10^−2^–9.3×10^−2^ substitutions/site/MY; [60]).

### Date estimation

Median coefficients of variation from the uncorrelated lognormal clock for each gene and subset are shown in Table 2 and as relative branch rates in Figure S1. In the case of *Orcinus*, there was a tendency towards very high values (>>1) in most individual genes, suggesting very high rate variation in *Orcinus*. The exceptions were *ND1*, *COX1*, *CYTB*, *CR*, complete mitogenome, and the informative gene subset (*COX1*, *ATP6*, *ND3*, *CYTB*), whose coefficients of variation ranged between 0.66 (0.01–2.09 95%HPD) for *ND1* and 1 (0.01–2.20 95%HPD) for *COX1*. This is strong evidence that most individual genes are unsuitable for date estimation within this timeframe. The mitogenome and the informative gene subset presented coefficients of variation in the lower range (0.46 (0.01–1 95%HPD) and 0.74 (0.01–1.37 95%HPD), respectively), indicating more clocklike behavior than for most single genes.

Contrary to the lack of clock-like behavior in *Orcinus*, nearly all Delphinidae data exhibited clock like rates with median coefficients of variation between 0.08 (0–0.23 95% HPD) for *ND4*, and 0.4 (0.12–0.82 95% HPD) for *12S-16S*. Nevertheless, an ANOVA used to compare date estimates between genes for every node in *Orcinus* and Delphinidae resulted in significant differences (P<0.05 for both sequence datasets).

Differences in TMRCA distributions between the mitogenome and individual genes and gene subsets are shown in Table 3. These results are shown for Transient killer whales in *Orcinus* and subfamily Globicephalinae in Delphinidae only, as these nodes displayed the most deviation among TMRCA estimates for each taxonomic group. The main observation is that individual genes consistently overestimated TMRCAs, whereas the informative gene subsets produced either slight overestimation (0.51 for the killer whales) or underestimation (0.44 for the Delphinidae). In both sequence datasets, the informative gene subsets provided the lowest over- or underestimation values.

**Table 3:** Median rate substitutions/site/MY and coefficient of variation for individual genes and gene subsets.

	*Orcinus*				Delphinidae			
	Median rate (10^−3^)	95% HPD	Median coefficient of variation	95% HPD	Median rate (10^−3^)	95% HPDI	Median coefficient of variation	95% HPD
***12S16S***	0.72	0.30–1.20	7.52	3.30–9.90	2.3	1.81–2.86	1.81–2.86	0.12–0.82
***ND1***	1.94	0.90–3.36	0.66	0.01–2.09	4.76	3.82–5.82	3.82–5.82	0–0.32
***ND2***	1.1	0.44–2.07	5.55	3.41–9.90	5.85	4.86–7.46	4.86–7.46	0–0.37
***COX1***	2.31	1.16–3.97	1	0.01–2.20	4.07	3.29–4.95	3.29–4.95	0–0.36
***COX2***	0.91	0.26–1.87	5.52	3.81–10.59	4.19	3.23–5.30	3.23–5.30	0–0.58
***ATP8***	1.67	0.27–4.05	6.41	4.90–10.96	5.21	3.48–7.22	3.48–7.22	0–0.57
***ATP6***	2.06	0.87–3.75	5.27	3.19–9.75	5.8	4.51–7.31	4.51–7.31	0–0.56
***COX3***	1.28	0.49–2.44	4.69	3.08–10.13	4.01	3.13–4.99	3.13–4.99	0–0.43
***ND3***	1.88	0.56–3.79	4.18	3.61–10.47	5.31	3.99–6.88	3.99–6.88	0–0.49
***ND4L***	1.06	0.17–2.58	5.54	4.13–10.42	5.16	3.65–7.00	3.65–7.00	0–0.75
***ND4***	1.36	0.60–2.41	5.58	3.08–10.11	4.63	3.82–5.51	3.82–5.51	0–0.23
***ND5***	1.69	0.82–2.83	4.91	2.88–9.47	5.08	4.23–5.97	4.23–5.97	0–0.28
***CYTB***	1.76	0.81–3.07	0.73	0.01–2.36	5.19	4.22–6.27	4.22–6.27	0–0.35
***CR***	4.44	2.32–7.58	0.3	0.00–1.00	6.08	4.47–7.48	4.47–7.48	0–0.43
***ND1, COX1, ND4***	-	-	-	-	4.51	3.89–5.18	3.89–5.18	0.15–0.48
***COX1, ATP6, ND3, CYTB***	2.37	1.20–3.80	0.74	0.01–1.37	-	-	-	-
**Mitogenome**	2.61	1.50–3.90	0.46	0.01–1.95	4.24	3.70–4.76	3.70–4.76	0–0.15

**Figure 4:**
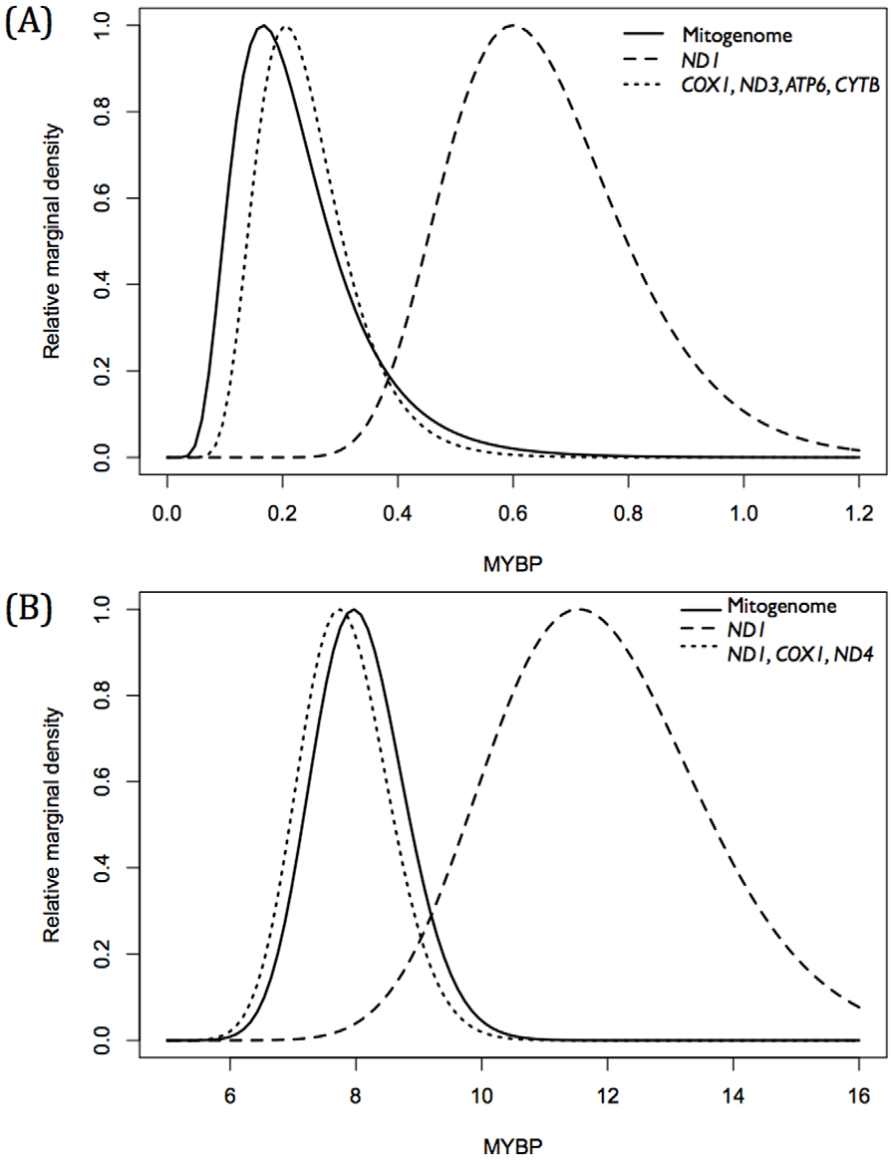
TMRCA distributions for the mitogenome, the most informative gene subsets and the gene providing the greatest overestimation for Transient killer whales (*Orcinus*) (A) and subfamily Globicephalinae (B). Note that time in MYBP is different for Transient killer whales and Globicephalinae.

Figure 4 shows the TMRCA distributions of the mitogenome, the informative gene subset, and the gene with largest TMRCA bias; *ND1* for *Orcinus* and *ND3* for Delphinidae. These genes overestimate median TMRCA by as much as 159% for Transient killer whales and 51% for Globicephalinae, in both cases producing broader distributions than either the mitogenome or the informative gene subset.

The accuracy of non-calibrated nodes in matching fossil calibrations was evaluated for Cetacea and Delphinidae based on sequential removal of internal node calibrations, allowing us to determine whether calibration priors were informative or not. In both sequence datasets, posterior distributions of TMRCAs contained the entirety of the distribution of the removed prior, so the proportion of the overlapping area under the curve of the removed prior within the posterior was 1 (i.e., the 95%HPD of the removed prior was contained within the posterior 95%HPD). This indicates that the remaining priors (eight in the Cetacea and two in Delphinidae) were capable of producing posterior TMRCAs that were similar to and completely consistent with the corresponding fossil calibration (see Table S4).

### Effects of partitioning on overall phylogenetic estimation

The four partitioning schemes used on the informative gene subsets revealed significant differences in TMRCA estimation among partitioning schemes for all nodes listed in Table 1, for both data sets. This was demonstrated by using an ANOVA and Tukey test for each node among schemes (P<0.004 for *Orcinus* and P<0.002 for Delphinidae, for all nodes). Nevertheless, the bias in TMRCA (from the mitogenomic estimate) only varied by 0.02 across schemes.

Analysis of the 95% credible set of trees of the four partitioning schemes revealed that all schemes for *Orcinus* and Delphinidae informative genes performed equally well in estimating the mitogenome topology. The mitogenomic tree was found within the credible set of trees for the delphinids but not for the killer whales, regardless of the partitioning scheme used.

Bayes factors suggested that the more partitioned models (12 partitions for *Orcinus* and 9 for Delphinidae) provided a better fit for both sequence datasets (lnBF = 150 (+/− 10) and 139 (+/− 5), using R and Tracer, respectively for *Orcinus*, and lnBF = 38(+/−4) and 30(+/−2) for Delphinidae). However, this may be an effect of using the harmonic mean, which appears to favor models with more parameters [63], [64].

## Discussion

This study provides an insight into the interpretation of mitogenomic phylogenetics through analyses of individual genes, subsets of genes, and the complete mitogenome. Complete mitogenomic results were considered to represent the highest phylogenetic performance and most reliable results compared to single genes and gene subsets. This rationale is based on the highly supported topologies, clock-like behavior, and low saturation displayed by the complete mitogenome, suggesting better-resolved trees and more precise date estimation than other partitions. Although we have focused on a timeframe of 11–0.5 MYBP and a limited taxonomic range (family to genus), our framework may be broadly applicable to other taxa across a variety of divergence times.

Testing for substitution models revealed similar evolutionary patterns in coding regions. *ND6* was an interesting exception, where a high bias towards transitions was observed but phylogenetic models failed to adequately account for this parameter (*Kappa*). Neglecting this estimate would likely lead to underparameterization. More complex models that can account for very high transition-transversion bias may facilitate inclusion of this region in future analyses.

Our main finding is that different regions of the mitogenome produced very different results, leading to incongruent topologies, poor PP clade support and conflicting date estimates. The low PP clade support in single gene topologies suggests insufficient informative variation for high phylogenetic resolution, implying a need for using larger portions of the mitogenome.

Informative subsets of genes (*ND1*, *COX1* and *ND4* for Delphinidae and *COX1*, *CYTB*, *ND3* and *ATP6* for *Orcinus*) were capable of summarizing the phylogenetic content of the complete mitogenome, indicating that information content in subsets of the mitogenome can be sufficient for phylogenetic analysis at a temporal scale below 15 MYBP, but that the choice of those subsets is taxon-dependent and might not be knowable prior to performing whole-mitogenome analysis on all taxa. At temporal scales beyond 15 MYBP, selection of fewer informative genes (e.g., *COX1*, *CYTB*, *ND1*) is likely to be sufficient, as a greater amount of variation will have accumulated.

There was also evidence of phylogenetic incongruence among individual gene analyses, indicated by the relatively long branches in Figure 3. This indicates that using single genes might not only produce low resolution but also different topologies. As the mitogenome is largely non-recombining, the evolutionary pattern of each gene should present an independent estimate of the evolution of the entire molecule. Incongruence in phylogenetic resolution can be explained by different substitution rates [16] or through different selective pressures across the mitogenome [65], [66].

All informative genes consisted of protein-coding regions, while *12S* and *16S* and the *CR* were found to be among the least informative. Possible explanations include very short variable regions in the *12S* and *16S* and mutational hotspots leading to early saturation in the *CR*, as has been suggested for human mtDNA [67]. Although we did not find evidence of saturation in the *CR* at shorter time scales, we would only expect to see this if it were evenly distributed across the region rather than at a few mutational hotspots. We suggest caution in phylogenetic studies based on this region, especially when complete mitogenomes or additional protein-coding regions are unavailable. As indicated by our results, the *CR* is likely to produce low resolution when estimating phylogenetic structure of taxonomic groups [20], especially in those recently diverged like *Orcinus*. More appropriate use of the *CR* is for population-level frequency differences in haplotypes.

An important aspect of topology and resolution in our results is that our use of highest clade credibility trees, rather than consensus trees, fails to take polytomies into account. Therefore genes with low resolution show trees with low PP support for particular clades, instead of showing polytomies. Distance between topologies estimated for Figure 3 would likely be different if majority-rule consensus trees were used instead of the maximum-clade-credibility tree. We consider our approach adequate as it allows distinction among the best gene trees, even if clades are weakly supported.

In order to address the potential for polytomies, we calculated the number of haplotypes detected by each gene compared to the whole mitogenome, whose underestimation would cause multiple haplotypes (as detected by the complete mitogenome) to collapse into a single taxon in the tree (Table S5). As expected, more conserved regions such as *ATP6* and *ATP8* detected few haplotypes, while more variable regions such as *CYTB* and the *CR* detected considerably more.

We draw some important conclusions concerning topologies and resolution: More conserved regions may provide low resolution (polytomies) while still containing valuable phylogenetic information, whereas highly variable regions can produce high resolution (fewer polytomies) for trees that do not reflect the topology of the complete mitogenome, as shown in Table 1. Moreover, genes that detect fewer haplotypes can still provide higher support for clades of interest, as was the case for *COX1*, compared to the *CR* in Delphinidae. This suggests that polytomies are within clades of interest and that more conserved regions can still produce better results (unless variability is extremely low, as in the *12S* and *16S*) than those that are highly variable.

Interestingly, the proportion of the complete mitogenome necessary to obtain mitogenome-level resolution in both taxonomic groups is very similar (3683 bp or 22.4% for Delphinidae, and 3518bp or 21.4% for *Orcinus*), even though they employed a different number of genes. A possible expected outcome was the necessity of more data for more recent timeframes; although more genes were necessary for *Orcinus*, the total sequence length was strikingly similar. Future studies aiming to capture mitogenome-level resolution may benefit from sequencing mitogenomes for a portion of the samples to determine the most informative genes, expected to cover a total sequence length of around 25% of the complete mitogenome for timeframes comparable to those used here. These genes alone can then be used for a broader sampling, effectively reducing sequencing time and cost.

Both gene subsets (for *Orcinus* and Delphinidae) shared *COX1* as a particularly informative gene. This is an important result considering its wide use as the marker of choice for DNA barcoding [8], [9], [68]. However, our results show that, though informative, the addition of more genes is necessary for systematics, notably in recently diverging (>1 MYBP) and taxonomically diverse groups.

Contrary to the low variation in substitution rates among genes in Delphinidae, informative genes in *Orcinus* (while not being the fastest) had relatively faster substitution rates than most other genes (Table 2), perhaps implying a relationship between phylogenetic information content and rate for these taxa and or this timeframe. Moreover, a key aspect of substitution rates is that extreme values correspond to very low information content such as in *12S-16S* and *COX2* with the lowest rates and the *CR* with the highest. This is likely explained by strong purifying selection in low variable regions and mutational hotspots and homoplasy [59] in those that are highly variable.

Date estimation and measures of clocklike behavior showed different patterns between Delphinidae and *Orcinus*. All genes in the Delphinidae were relatively clock-like, whereas in the killer whales, molecular clocks for most genes presented very high among-lineage rate variation. It could be assumed that this is due to highly variable rates in recent timeframes or within this taxon, but there is lower overall variation in killer whales, resulting in very few substitutions in most genes, and therefore higher inferred rate heterogeneity among lineages with very few or no variable sites.

It has been suggested that faster rates are observed for more recent timeframes and that the trend disappears after 1 MY [69], so our finding that the rate of the killer whales is faster than that in delphinids may be accurate. We therefore conclude that our thorough sampling of killer whales and adequate calibrations produce reliable rate estimates, and that the *CR* has a rate similar to previous reports for these taxa.

Clock-like behavior was not linked to tree topology or information content. *ATP6* and *ND3* on their own (and in partitioning strategies where a separate clock is assumed for either of them) presented substantial rate heterogeneity, but they provided key phylogenetic information (*ND3* being the only gene to support monophyly for the Antarctic type C clade (AntC) and *ATP6* one of the few to support the Resident clade). On the other hand, very low rate heterogeneity was observed for the *CR*, yet it provided no support for six of the eight *Orcinus* clades. As a result, when choosing gene subsets and partitioning strategies one must take into account that high levels of variation and clocklike behavior do not automatically produce high phylogenetic resolution, and genes with high rate heterogeneity (in this case due to low variation) can still provide key information for taxonomic and evolutionary studies.

Despite the inferred rate heterogeneity among loci for the killer whale dataset, individual genes always produced overestimates of TMRCAs, which was considerably reduced by adding more genes in the gene subset analyses. Considerable differences in date and rate estimation are common among studies in cetaceans, notably in the killer whales [see 17,31,32]. Choice of calibrations (priors) will have a direct effect, and addressing this issue requires strong paleontological constraints on molecular clocks. However, differences in these estimates can also be expected depending on the sequences used, as we have shown. Reliable date estimation that reflects the whole mitogenome sequence analysis is unachievable using single markers, even while using identical calibrations. Combining genes through supermatrix methods, simple concatenation or species trees are more reliable approaches [14], [70].

Reliability of calibrations and their fit to the molecular clock in the cetacean and Delphinidae phylogenies showed that every excluded calibration prior not used could be estimated by the those remaining, meaning that excluded calibration priors were not informative, and amounting to two independent lines of evidence on divergence times in Cetacea (molecular estimates and fossil-based calibrations). Moreover, this is supportive of the overall concordance of the calibrations, the data and the phylogenetic models, so estimates such as TMRCA and substitution rates are accurate [55] and in agreement with previous estimates using the same calibrations [14], [18]. Further insights on date estimation reliability and its concordance to paleontologic or geologic evidence must take into account how well calibrations are distributed along the phylogeny. New clock and phylogenetic models can provide better frameworks for molecular dating, for example taking into account rate changes along lineages [71], accounting for rate heterogeneity among sites[16] and multi-gene approaches [72], [73].

Biologically sound partitioning strategies (e.g., using codon positions) applied to the most informative gene subsets showed no effect in producing more mitogenome-like results in terms of topologies and divergence times. This may be a consequence of little difference in substitution models and rates among the genes used. When incorporating mitochondrial regions with different substitution models and selective constraints, partitioning by substitution models and clocks may result in an improvement in quality of results and likelihood [16]. However using the informative genes only, precise and accurate estimation can be obtained without the need for partitioning.

Better estimators for the marginal likelihood (such as thermodynamic integration) may better evaluate partitioning strategies by not favoring overparameterized models [63], [64]. These are difficult to employ and largely unavailable at this time, but other non-parametric methods, like site shuffling [74], can add statistical reliability to these tests for partitioning strategies.

Beyond specific results, we present an overview of approaches to mitochondrial phylogenetics and suggest the following for future research:

- Topologies from individual genes are likely to differ because of different substitution rates. However, analyses should not seek consensus approaches from individual gene trees since these would result in largely unresolved trees due to their variability in topologies; rather, constraining all the data to a single tree will produce better results. This can be achieved through concatenation (either partitioned or un-partitioned) or using species tree approaches where genes share a unique tree prior.
- When informative genes (in terms of topologies) have high coefficients of variation (often due to low sequence variability), partitioned models that assign separate substitution and clock models to these regions will produce unreliable date estimation, and a non-partitioned approach will work best. These partitioning parameters should be taken into account in addition to likelihood-based tests for choosing a partitioning strategy.
- A minimum number of genes can be used, capable of reproducing mitogenomic results to optimize large analyses or use datasets with incomplete mitogenomic sequences. The most informative loci are likely the coding regions and can amount to ∼25% of the complete mitogenome, although this is subject to vary depending on the taxonomic group. Apart from standard deviations from the molecular clock, it is important to assess how many clades of interest each gene supports to avoid loss of information. Gene subsets meeting these requirements should reliably estimate divergence times and phylogenetic relationships comparable to results for the entire mitogenome.
- Saturation should be taken into account when choosing genes for informative gene subsets or removing uninformative data from mitochondrial genomes. The control region is of special concern given its popularity. Our results showed this was a highly variable region but provided low phylogenetic resolution and overestimation of divergence times. This is probably due to mutational hotspots, therefore variation is not equivalent to information content.
- Single gene analyses tend to produce consistent overestimation of divergence times compared to the mitogenome, and thus should be avoided. Date estimation is most accurate with gene subsets or complete mitochondrial genomes.
- Previous studies based on single mitochondrial markers can benefit from adding more informative genes. In our examples, use of the entire mitogenome produced highly robust and consistent results; displaying the highest PP for clades of interest among partitions, clock-like behavior, precise date estimation (narrow highest posterior density intervals) and low saturation.

## Supporting Information

Figure S1 **Chronogram trees for complete mitogenomes and partitions analyzed.** Shades of blue in branches represent relative substitution rates along the tree so that trees with wide range of shades have higher rate heterogeneity. Light blue suggests slower rates than darker shades (faster rates).
(PDF)

Figure S2 **Saturation plot of standardized distances for the cetacean dataset.** Red: Control region, Black: Complete mitogenome, Blue: Informative gene subsets for *Orcinus* and Delphinidae.
(TIFF)

Table S1 **Species and haplotype names, paper reference and GenBank Accession number.**
(DOC)

Table S2 **Prior distributions used as calibrations for phylogenetic analyses as estimated by Morin et al.** 2010 for all sequence Datasets. Values correspond to ages in mya after log transformation. mya  =  Million years ago. *Calibration priors for Orcinus and Delphinidae sequence datasets correspond to posterior distributions estimated from complete mitogenomic analysis of a Cetacean sequence dataset by Morin et al (2010) and confirmed in this study. Calibrations sequentially removed in “Accuracy of TMRCA estimates for non-calibrated nodes” section for the Cetacea and Delphinidae.
(DOC)

Table S3 **Substitution models used according to the BIC.**
(DOCX)

Table S4 **Posterior TMRCA estimates for nodes in** ***Orcinus*** **, Delphinidae and Cetacea datasets using the complete mitogenome.** Dates are expressed in Million years before present (MYBP).
(DOCX)

Table S5 **Proportion of unique haplotypes detected by each gene for both datasets, alignment length and % similarity.** Note that intraspecific sampling coverage is not equivalent between datasets so comparisons of the number of haplotypes for a given gene is not appropriate.
(DOCX)
