# Supplementary material for: Mitogenome Phylogenetics: The Impact of Using Single Regions and Partitioning Schemes on Topology, Substitution Rate and Divergence Time Estimation
Source: PLoS One. 2011 Nov 2;6(11):e27138. doi: 10.1371/journal.pone.0027138 (PMC3206919; doi:10.1371/journal.pone.0027138)
Supplement: Figure S1 — Chronogram trees for complete mitogenomes and partitions analyzed. Shades of blue in branches represent relative substitution rates along the tree so that trees with wide range of shades have higher rate heterogeneity. Light blue suggests slower rates than darker shades (faster rates). (PDF) [file pone.0027138.s001.pdf]

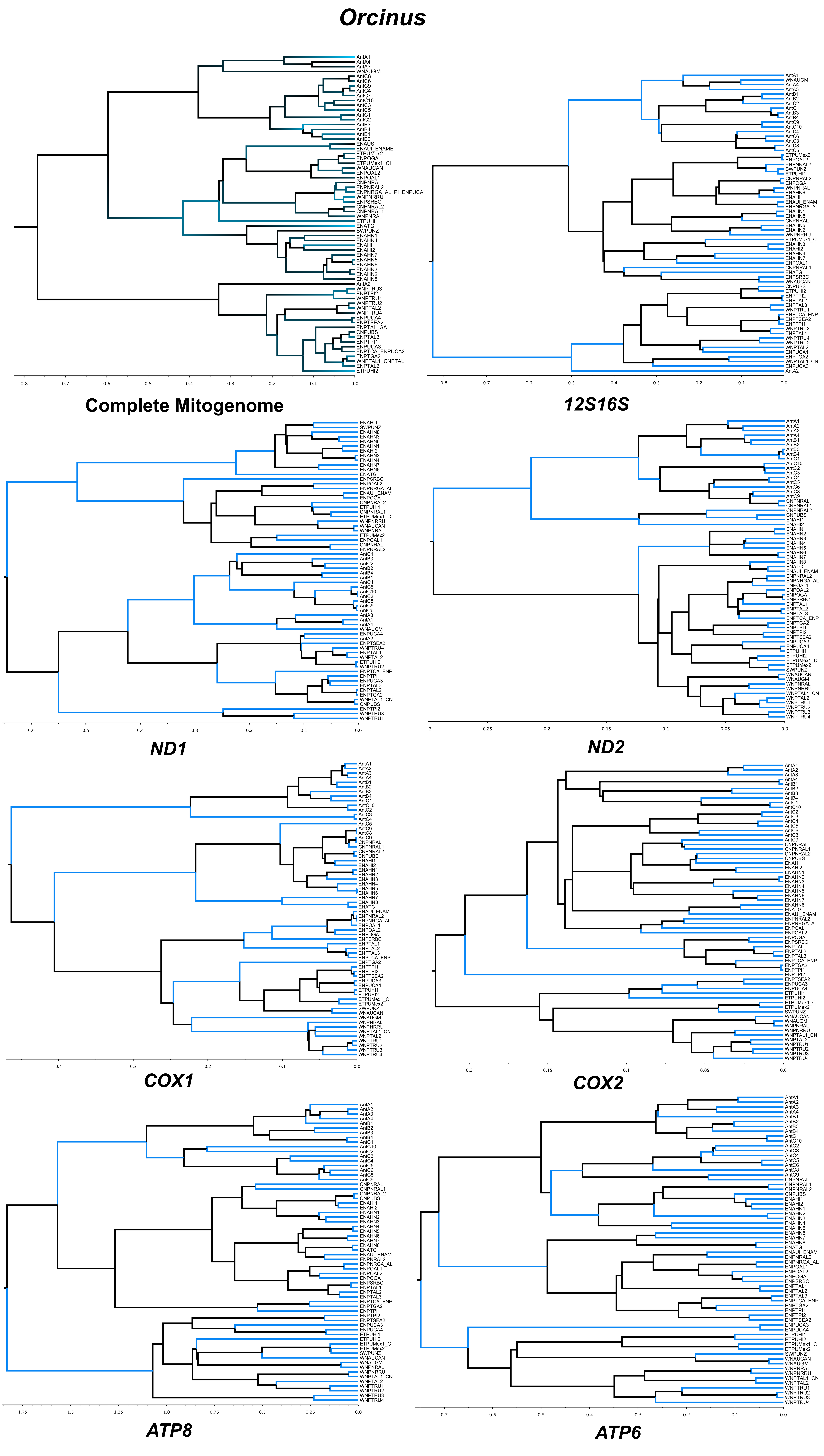
Orcinus
Complete Mitogenome
12S16S
ND1
ND2
COX1
COX2
ATP8
ATP6

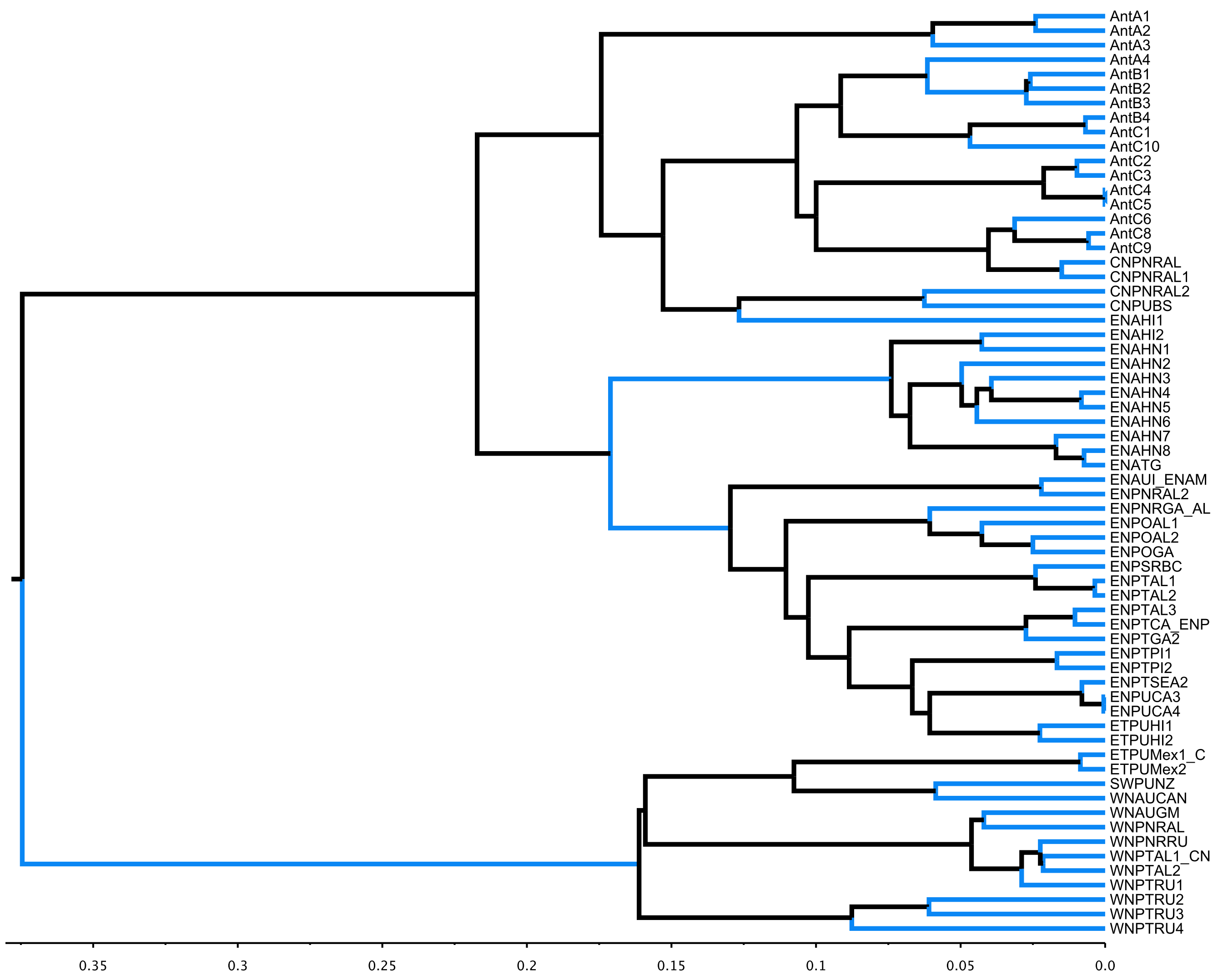

COX3

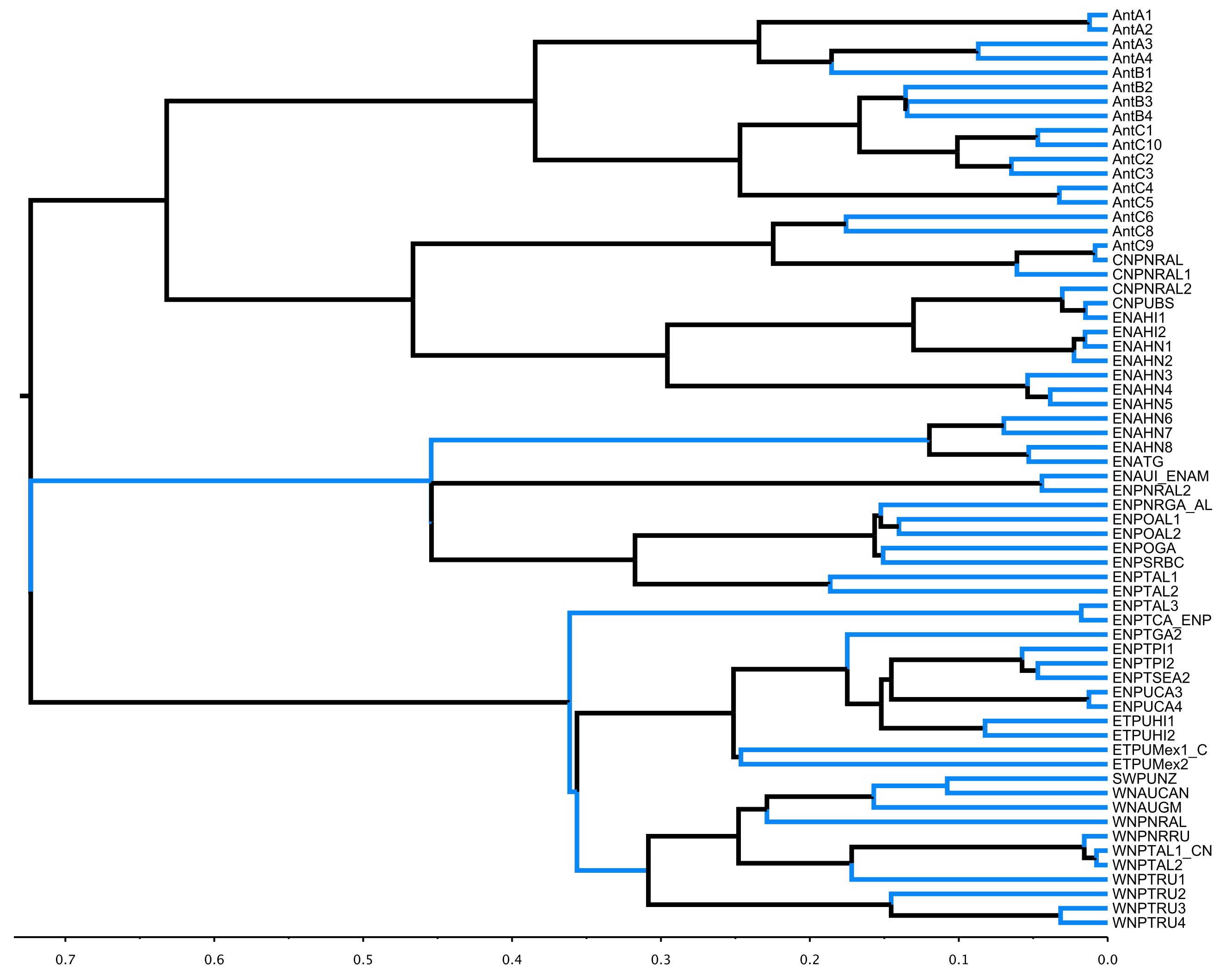

ND3

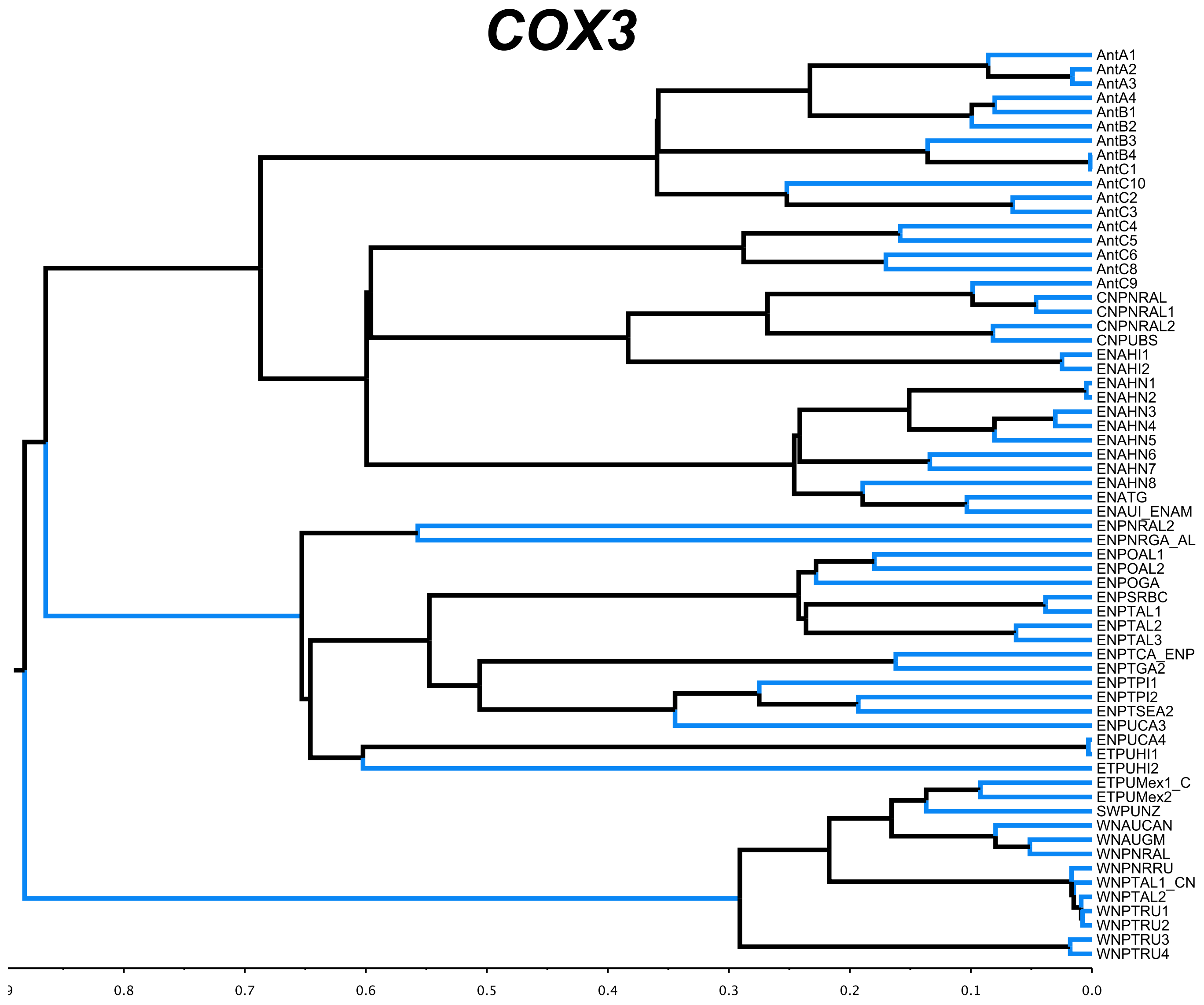

ND4L

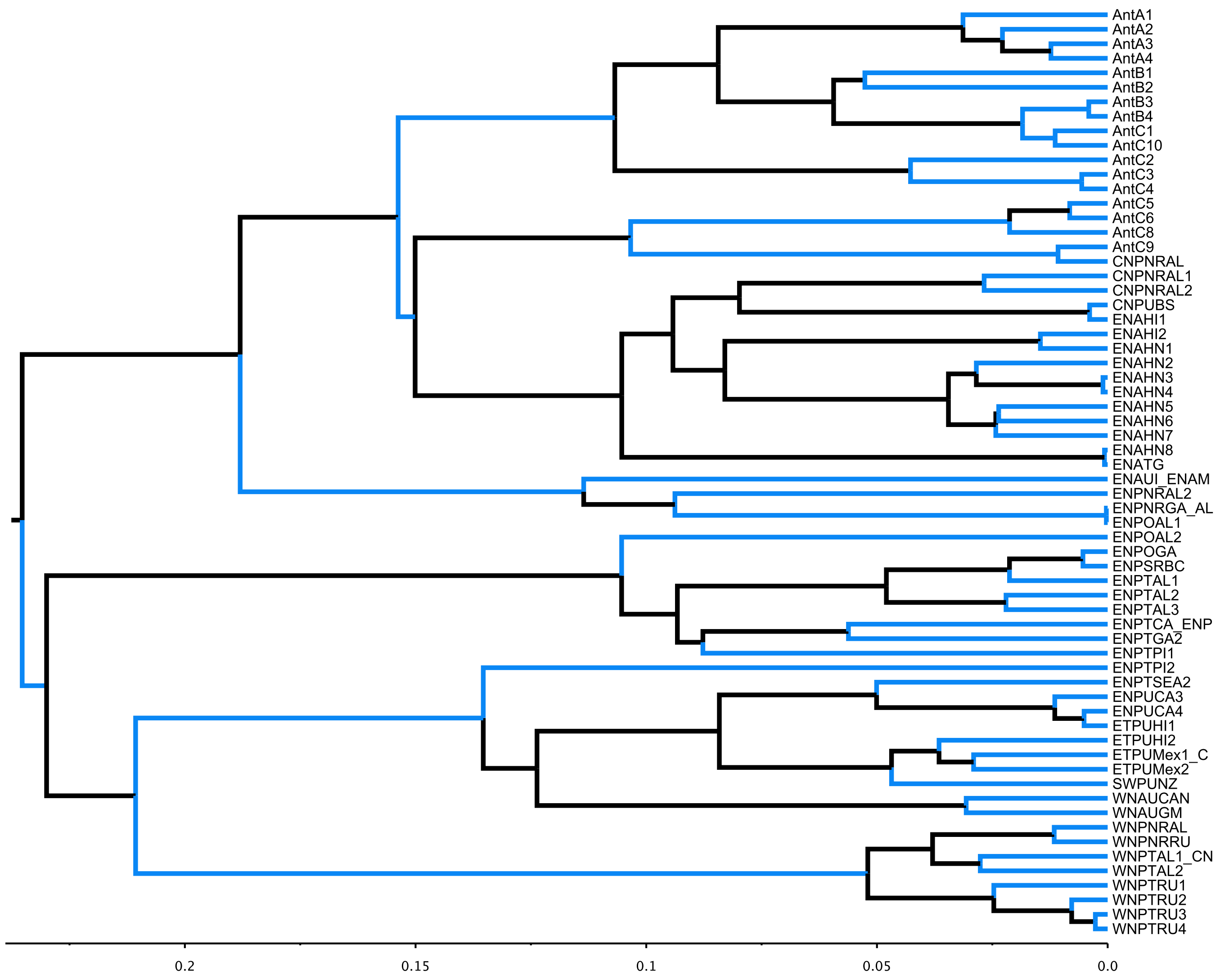

ND4

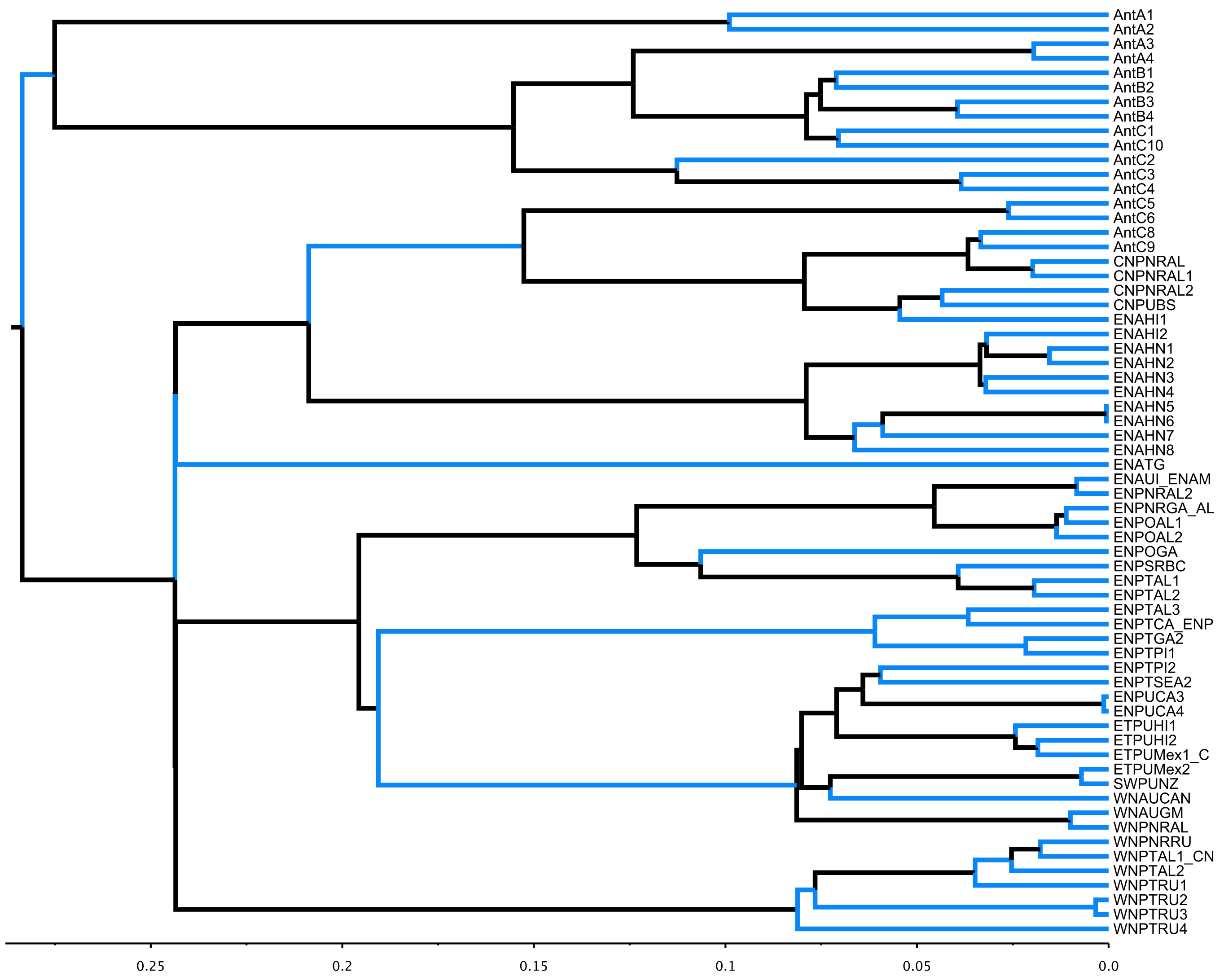

ND5

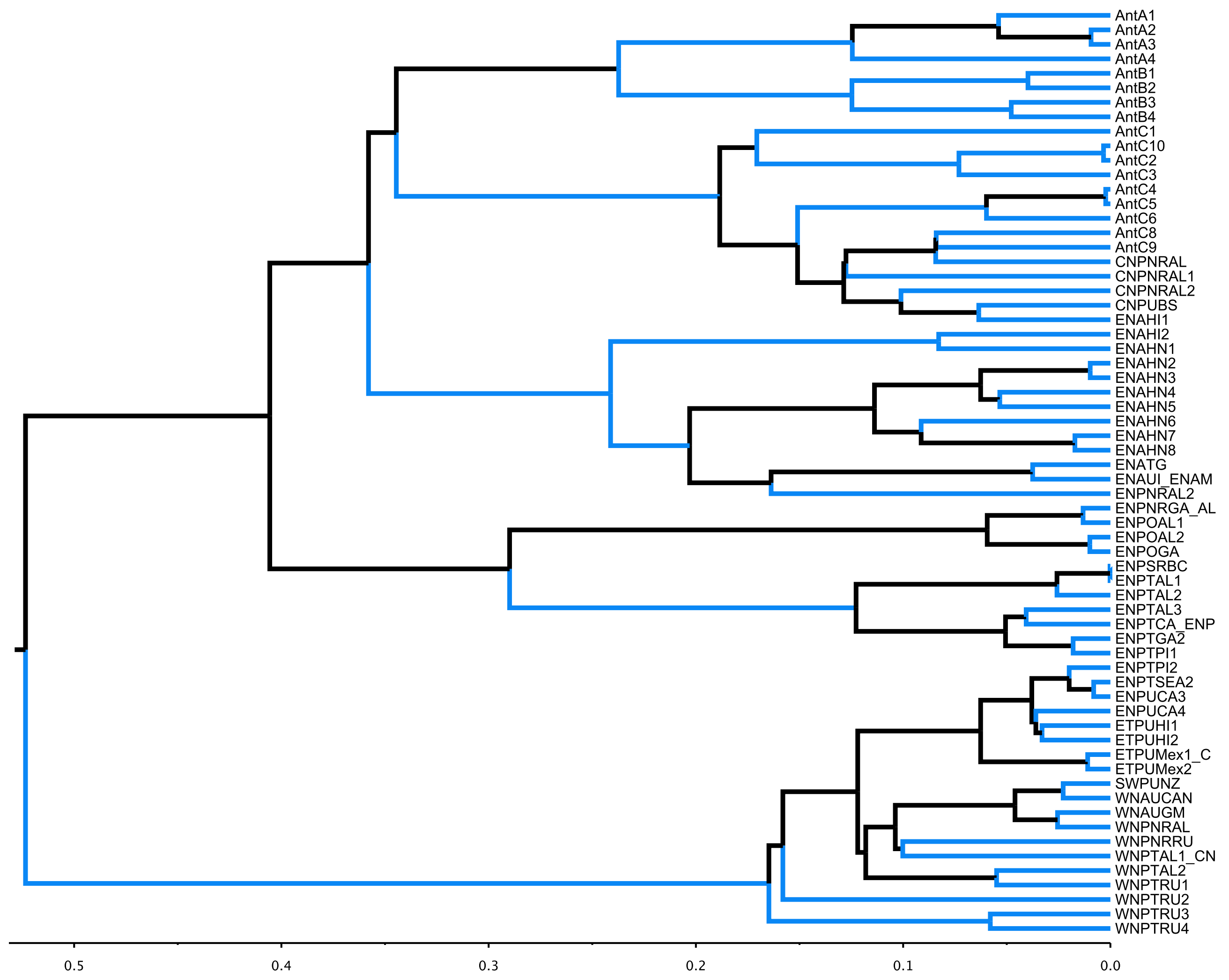

CYTB

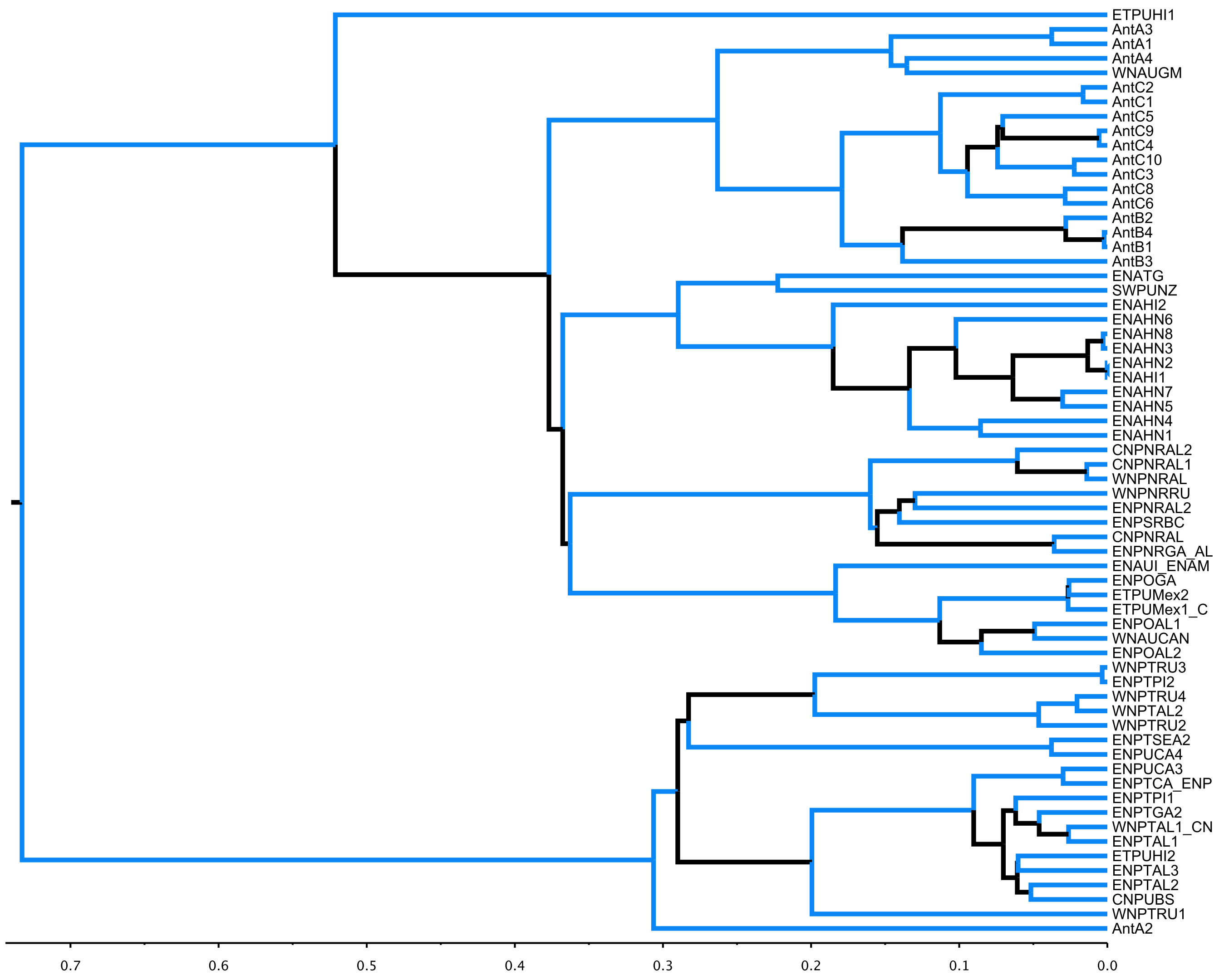

CR

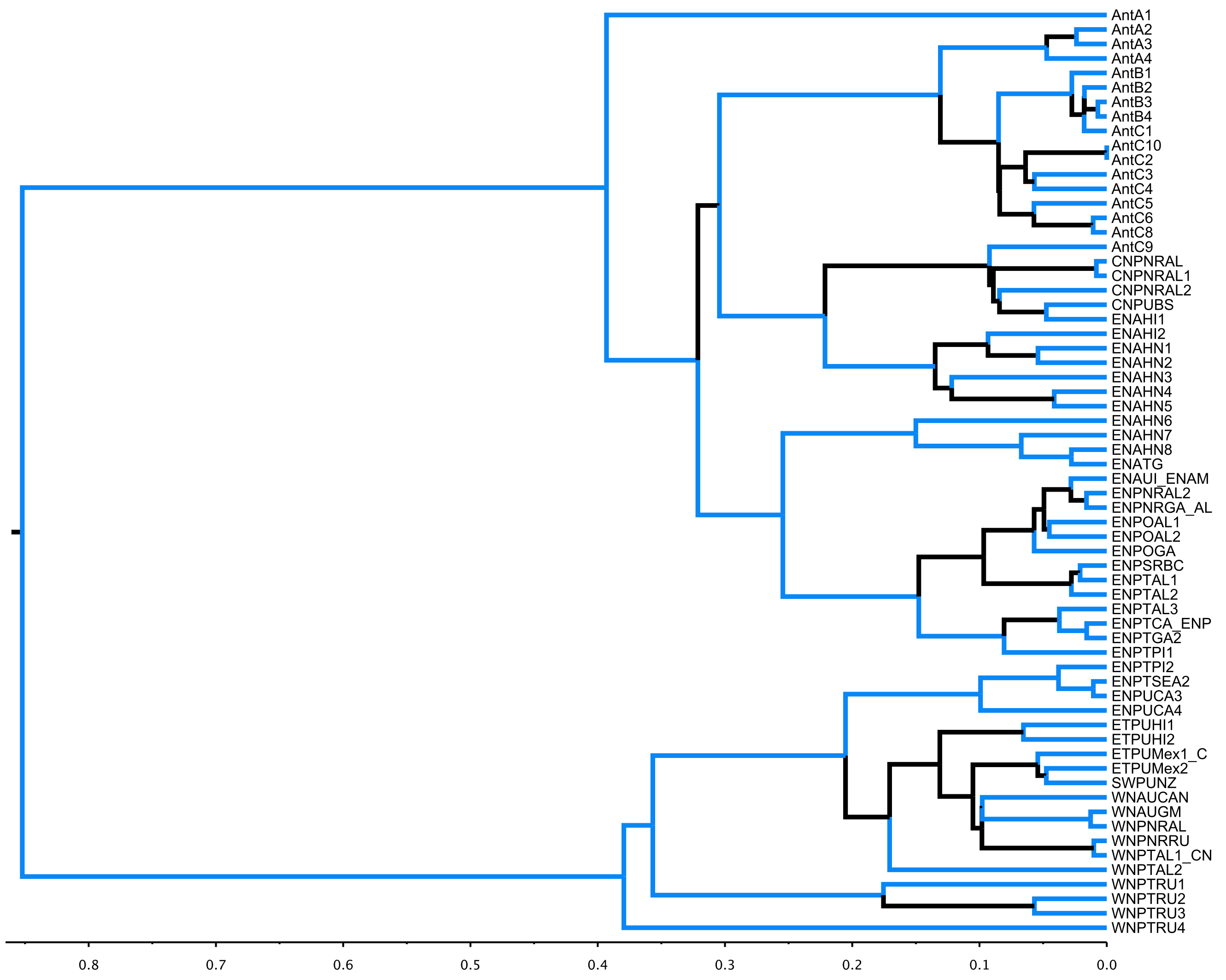

COX1, ATP6, ND3, CYTB

Delphinidae

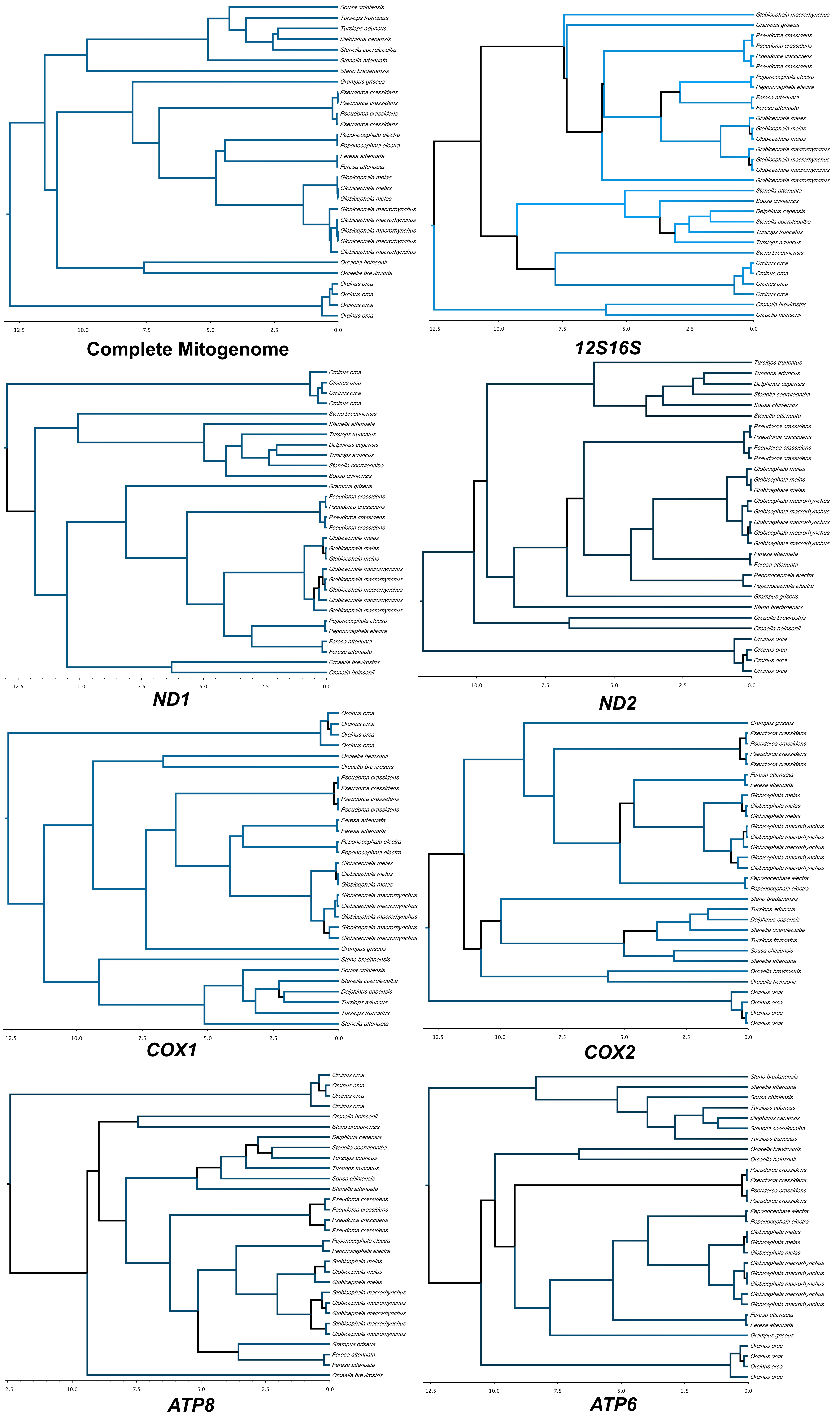

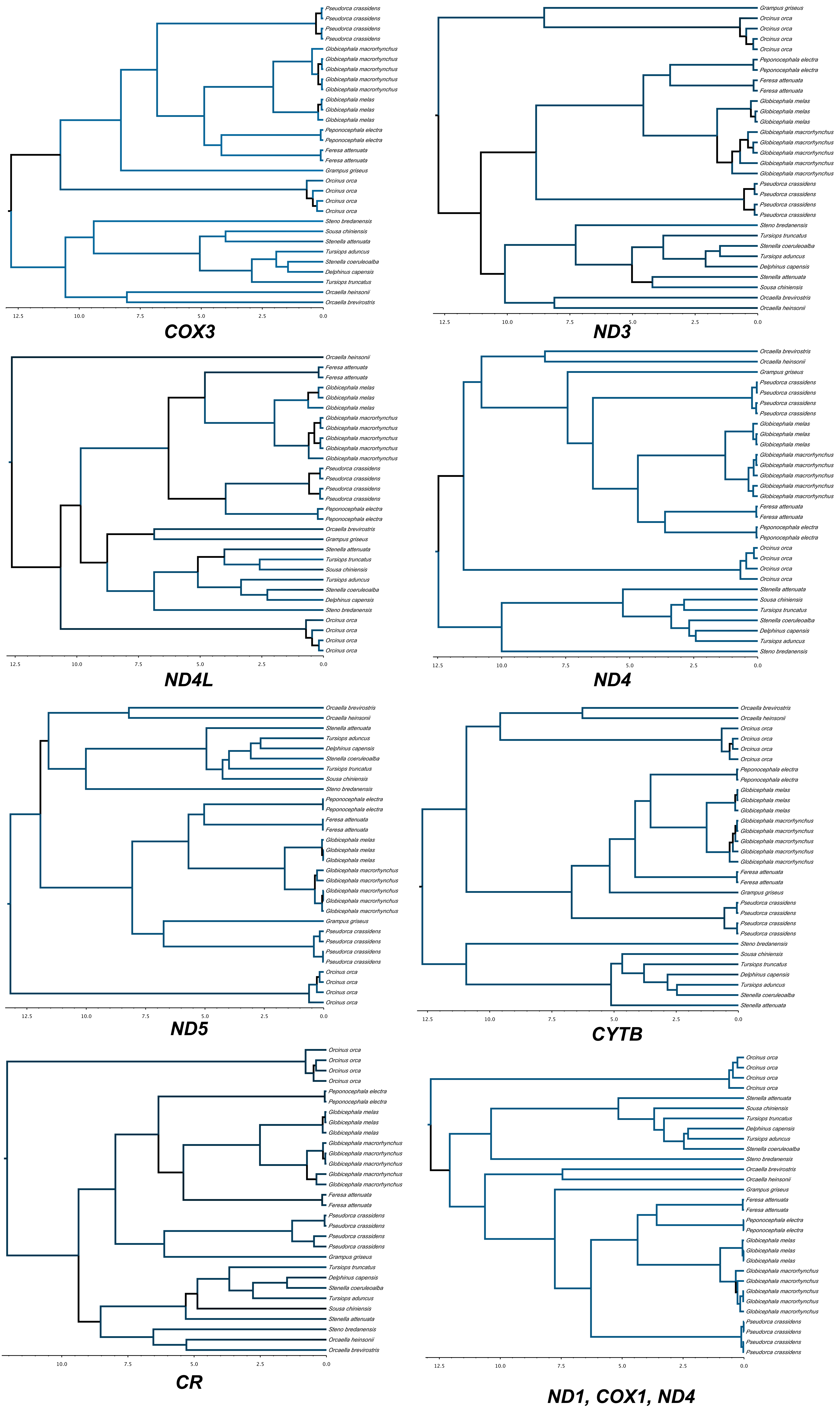

Pseudorca crassidens
Pseudorca crassidens
Pseudorca crassidens
Pseudorca crassidens
Globicephala macrorhynchus
Globicephala macrorhynchus
Globicephala macrorhynchus
Globicephala macrorhynchus
Globicephala macrorhynchus
Globicephala melas
Globicephala melas
Globicephala melas
Peponocephala electra
Peponocephala electra
Feresa attenuata
Feresa attenuata
Grampus griseus
Orcinus orca
Orcinus orca
Orcinus orca
Orcinus orca
Steno bredanensis
Sousa chiniensis
Stenella attenuata
Tursiops aduncus
Stenella coeruleoalba
Delphinus capensis
Tursiops truncatus
Orcaella heinsonii
Orcaella brevirostris
12.5
10.0
7.5
5.0
2.5
0.0
COX3
Grampus griseus
Orcinus orca
Orcinus orca
Orcinus orca
Orcinus orca
Peponocephala electra
Peponocephala electra
Feresa attenuata
Feresa attenuata
Globicephala melas
Globicephala melas
Globicephala melas
Globicephala macrorhynchus
Globicephala macrorhynchus
Globicephala macrorhynchus
Globicephala macrorhynchus
Globicephala macrorhynchus
Pseudorca crassidens
Pseudorca crassidens
Pseudorca crassidens
Pseudorca crassidens
Steno bredanensis
Tursiops truncatus
Stenella coeruleoalba
Tursiops aduncus
Delphinus capensis
Stenella attenuata
Sousa chiniensis
Orcaella brevirostris
Orcaella heinsonii
12.5
10.0
7.5
5.0
2.5
0.0
ND3
Orcaella heinsonii
Feresa attenuata
Feresa attenuata
Globicephala melas
Globicephala melas
Globicephala melas
Globicephala macrorhynchus
Globicephala macrorhynchus
Globicephala macrorhynchus
Globicephala macrorhynchus
Globicephala macrorhynchus
Pseudorca crassidens
Pseudorca crassidens
Pseudorca crassidens
Pseudorca crassidens
Peponocephala electra
Peponocephala electra
Orcaella brevirostris
Grampus griseus
Stenella attenuata
Tursiops truncatus
Sousa chiniensis
Tursiops aduncus
Stenella coeruleoalba
Delphinus capensis
Steno bredanensis
Orcinus orca
Orcinus orca
Orcinus orca
Orcinus orca
12.5
10.0
7.5
5.0
2.5
0.0
ND4L
Orcaella brevirostris
Orcaella heinsonii
Grampus griseus
Pseudorca crassidens
Pseudorca crassidens
Pseudorca crassidens
Pseudorca crassidens
Globicephala melas
Globicephala melas
Globicephala melas
Globicephala macrorhynchus
Globicephala macrorhynchus
Globicephala macrorhynchus
Globicephala macrorhynchus
Globicephala macrorhynchus
Feresa attenuata
Feresa attenuata
Peponocephala electra
Peponocephala electra
Orcinus orca
Orcinus orca
Orcinus orca
Orcinus orca
Stenella attenuata
Sousa chiniensis
Tursiops truncatus
Stenella coeruleoalba
Delphinus capensis
Tursiops aduncus
Steno bredanensis
12.5
10.0
7.5
5.0
2.5
0.0
ND4
Orcaella brevirostris
Orcaella heinsonii
Stenella attenuata
Tursiops aduncus
Delphinus capensis
Stenella coeruleoalba
Tursiops truncatus
Sousa chiniensis
Steno bredanensis
Peponocephala electra
Peponocephala electra
Feresa attenuata
Feresa attenuata
Globicephala melas
Globicephala melas
Globicephala melas
Globicephala macrorhynchus
Globicephala macrorhynchus
Globicephala macrorhynchus
Globicephala macrorhynchus
Globicephala macrorhynchus
Grampus griseus
Pseudorca crassidens
Pseudorca crassidens
Pseudorca crassidens
Pseudorca crassidens
Orcinus orca
Orcinus orca
Orcinus orca
Orcinus orca
12.5
10.0
7.5
5.0
2.5
0.0
ND5
Orcaella brevirostris
Orcaella heinsonii
Orcinus orca
Orcinus orca
Orcinus orca
Orcinus orca
Peponocephala electra
Peponocephala electra
Globicephala melas
Globicephala melas
Globicephala melas
Globicephala macrorhynchus
Globicephala macrorhynchus
Globicephala macrorhynchus
Globicephala macrorhynchus
Globicephala macrorhynchus
Feresa attenuata
Feresa attenuata
Grampus griseus
Pseudorca crassidens
Pseudorca crassidens
Pseudorca crassidens
Pseudorca crassidens
Steno bredanensis
Sousa chiniensis
Tursiops truncatus
Delphinus capensis
Tursiops aduncus
Stenella coeruleoalba
Stenella attenuata
12.5
10.0
7.5
5.0
2.5
0.0
CYTB
Orcinus orca
Orcinus orca
Orcinus orca
Orcinus orca
Peponocephala electra
Peponocephala electra
Globicephala melas
Globicephala melas
Globicephala melas
Globicephala macrorhynchus
Globicephala macrorhynchus
Globicephala macrorhynchus
Globicephala macrorhynchus
Globicephala macrorhynchus
Feresa attenuata
Feresa attenuata
Pseudorca crassidens
Pseudorca crassidens
Pseudorca crassidens
Pseudorca crassidens
Grampus griseus
Tursiops truncatus
Delphinus capensis
Stenella coeruleoalba
Tursiops aduncus
Sousa chiniensis
Stenella attenuata
Steno bredanensis
Orcaella heinsonii
Orcaella brevirostris
10.0
7.5
5.0
2.5
0.0
CR
Orcinus orca
Orcinus orca
Orcinus orca
Orcinus orca
Stenella attenuata
Sousa chiniensis
Tursiops truncatus
Delphinus capensis
Tursiops aduncus
Stenella coeruleoalba
Steno bredanensis
Orcaella brevirostris
Orcaella heinsonii
Grampus griseus
Feresa attenuata
Feresa attenuata
Peponocephala electra
Peponocephala electra
Globicephala melas
Globicephala melas
Globicephala melas
Globicephala macrorhynchus
Globicephala macrorhynchus
Globicephala macrorhynchus
Globicephala macrorhynchus
Globicephala macrorhynchus
Pseudorca crassidens
Pseudorca crassidens
Pseudorca crassidens
Pseudorca crassidens
12.5
10.0
7.5
5.0
2.5
0.0
ND1, COX1, ND4
