# Supplementary figures and images for: Mitogenome Phylogenetics: The Impact of Using Single Regions and Partitioning Schemes on Topology, Substitution Rate and Divergence Time Estimation
Source: PLoS One. 2011 Nov 2;6(11):e27138. doi: 10.1371/journal.pone.0027138 (PMC3206919; doi:10.1371/journal.pone.0027138)

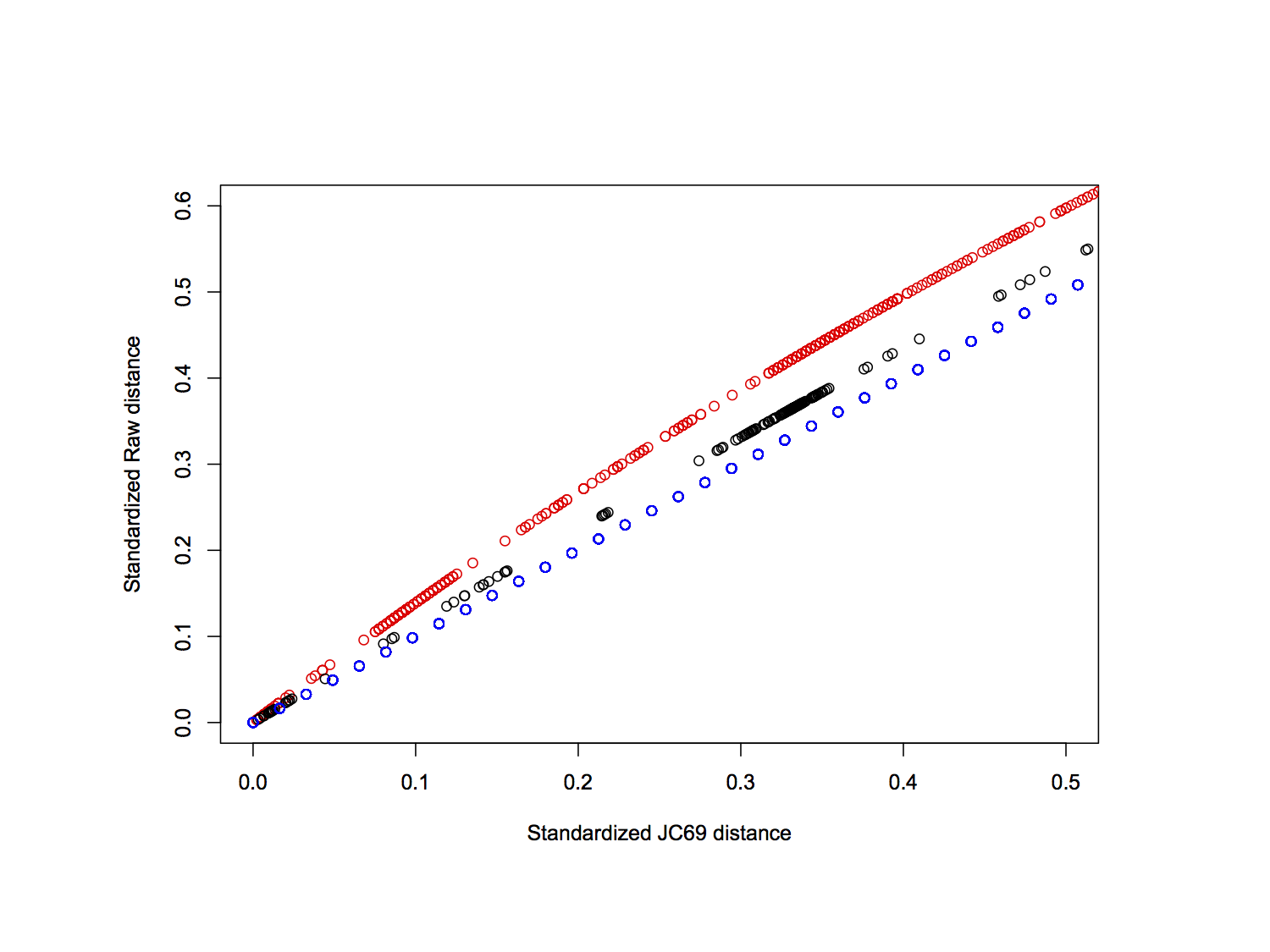

Supplement: Figure S2 — Saturation plot of standardized distances for the cetacean dataset. Red: Control region, Black: Complete mitogenome, Blue: Informative gene subsets for Orcinus and Delphinidae. (TIFF) [file pone.0027138.s002.tif]
