# Supplementary material for: Mitogenome Phylogenetics: The Impact of Using Single Regions and Partitioning Schemes on Topology, Substitution Rate and Divergence Time Estimation
Source: PLoS One. 2011 Nov 2;6(11):e27138. doi: 10.1371/journal.pone.0027138 (PMC3206919; doi:10.1371/journal.pone.0027138)
Supplement: Table S1 — Species and haplotype names, paper reference and GenBank Accession number. (DOC) [file pone.0027138.s003.doc]

**Table S1.** Species and haplotype names, paper reference and GenBank Accession number.

| **Species name** | | **Reference** | | **ACCN** | |
| --- | --- | --- | --- | --- | --- |
| ***Balaena mysticetus*** |  | | Arnason et al. 2004 | | **NC005268** |
| ***Balaenoptera acutorostrata*** |  | | Arnason et al. 2004 | | **NC005271** |
| ***Balaenoptera musculus*** |  | | Arnason et al. 2004 | | **NC001601** |
| ***Balaenoptera physalus*** |  | | Arnason et al. 2004 | | **NC001321** |
| ***Caperea marginata*** |  | | Arnason et al. 2004 | | **NC005269** |
| ***Delphinus capensis*** |  | | Xiong et al. 2009 | | **EU557094** |
| ***Delphinus capensis*** |  | | Xiong et al. 2009 | | **EU557094** |
| ***Eschrichtius robustus*** |  | | Arnason et al. 2004 | | **NC005270** |
| ***Feresa attenuata*** |  | | Vilstrup et al. 2011 | | **JF289172** |
| ***Feresa attenuata*** |  | | Vilstrup et al. 2011 | | **JF289171** |
| ***Globicephala macrorhynchus*** |  | | Vilstrup et al. 2011 | | **JF339974** |
| ***Globicephala macrorhynchus*** |  | | Vilstrup et al. 2011 | | **JF339976** |
| ***Globicephala macrorhynchus*** |  | | Morin et al. 2010 | | **HM060334** |
| ***Globicephala macrorhynchus*** |  | | Vilstrup et al. 2011 | | **JF339975** |
| ***Globicephala melas*** |  | | Morin et al. 2010 | | **HM060334** |
| ***Globicephala melas*** |  | | Vilstrup et al. 2011 | | **JF339972** |
| ***Grampus griseus*** |  | | Xiong et al. 2009 | | **EU557095** |
| ***Grampus grisseus*** |  | | Xiong et al. 2009 | | **EU557095** |
| ***Hyperoodon ampullatus*** |  | | Arnason et al. 2004 | | **NC005273** |
| ***Inia geoffrensis*** |  | | Arnason et al. 2004 | | **NC005276** |
| ***Kogia breviceps*** |  | | Arnason et al. 2004 | | **NC005272** |
| ***Lagenorhynchus albirostris*** |  | | Arnason et al. 2004 | | **NC005278** |
| ***Lipotes vexillifer*** |  | | Yan et al. 2005 | | **NC007629** |
| ***Megaptera novaeanglia*** |  | | Sasaki et al. 2005 | | **NC006927** |
| ***Monodon monoceros*** |  | | Arnason et al. 2004 | | **NC005279** |
| ***Orcaella brevirostris*** |  | | Vilstrup et al. 2011 | | **JF289177** |
| ***Orcaella heinsoni*** |  | | Vilstrup et al. 2011 | | **JF339977** |
| ***Orcinus orca*** | AntA1 | | Morin et al. 2010 | | **GU187217** |
| ***Orcinus orca*** | AntA4 | | Morin et al. 2010 | | **GU187219** |
| ***Orcinus orca*** | AntA3 | | Morin et al. 2010 | | **GU187218** |
| ***Orcinus orca*** | AntC8 | | Morin et al. 2010 | | **GU187216** |
| ***Orcinus orca*** | AntC6 | | Morin et al. 2010 | | **GU187203** |
| ***Orcinus orca*** | AntC9 | | Morin et al. 2010 | | **GU187206** |
| ***Orcinus orca*** | AntC4 | | Morin et al. 2010 | | **GU187205** |
| ***Orcinus orca*** | AntC7 | | Morin et al. 2010 | | **GU187153** |
| ***Orcinus orca*** | AntC10 | | Morin et al. 2010 | | **GU187209** |
| ***Orcinus orca*** | AntC3 | | Morin et al. 2010 | | **GU187207** |
| ***Orcinus orca*** | AntC5 | | Morin et al. 2010 | | **GU187208** |
| ***Orcinus orca*** | AntC1 | | Morin et al. 2010 | | **GU187210** |
| ***Orcinus orca*** | AntC2 | | Morin et al. 2010 | | **GU187211** |
| ***Orcinus orca*** | AntB3 | | Morin et al. 2010 | | **GU187212** |
| ***Orcinus orca*** | AntB1 | | Morin et al. 2010 | | **GU187214** |
| ***Orcinus orca*** | AntB2 | | Morin et al. 2010 | | **GU187212** |
| ***Orcinus orca*** | ENAUS | | Morin et al. 2010 | | **GU187213** |
| ***Orcinus orca*** | ENAUI_ENAME | | Morin et al. 2010 | | **GU187188** |
| ***Orcinus orca*** | ETPUMex2 | | Morin et al. 2010 | | **GU187198** |
| ***Orcinus orca*** | ENPOGA | | Morin et al. 2010 | | **GU187197** |
| ***Orcinus orca*** | ETPUMex1_CI | | Morin et al. 2010 | | **GU187199** |
| ***Orcinus orca*** | WNAUCAN | | Morin et al. 2010 | | **GU187201** |
| ***Orcinus orca*** | ENPOAL2 | | Morin et al. 2010 | | **GU187200** |
| ***Orcinus orca*** | CNPNRAL | | Morin et al. 2010 | | **GU187189** |
| ***Orcinus orca*** | ENPNRAL2 | | Morin et al. 2010 | | **GU187194** |
| ***Orcinus orca*** | ENPNRGA_AL_PI_ENPUCA1 | | Morin et al. 2010 | | **GU187193** |
| ***Orcinus orca*** | WNPNRRU | | Morin et al. 2010 | | **GU187196** |
| ***Orcinus orca*** | ENPSRBC | | Morin et al. 2010 | | **GU187195** |
| ***Orcinus orca*** | CNPNRAL2 | | Morin et al. 2010 | | **GU187191** |
| ***Orcinus orca*** | CNPNRAL1 | | Morin et al. 2010 | | **GU187190** |
| ***Orcinus orca*** | WNPNRAL | | Morin et al. 2010 | | **GU187192** |
| ***Orcinus orca*** | ETPUHI1 | | Morin et al. 2010 | | **GU187187** |
| ***Orcinus orca*** | ENATG | | Morin et al. 2010 | | **GU187176** |
| ***Orcinus orca*** | SWPUNZ | | Morin et al. 2010 | | **GU187175** |
| ***Orcinus orca*** | ENAHN1 | | Morin et al. 2010 | | **GU187178** |
| ***Orcinus orca*** | ENAHN4 | | Morin et al. 2010 | | **GU187177** |
| ***Orcinus orca*** | ENAHI1 | | Morin et al. 2010 | | **GU187180** |
| ***Orcinus orca*** | ENAHI2 | | Morin et al. 2010 | | **GU187179** |
| ***Orcinus orca*** | ENAHN7 | | Morin et al. 2010 | | **GU187185** |
| ***Orcinus orca*** | ENAHN5 | | Morin et al. 2010 | | **GU187186** |
| ***Orcinus orca*** | ENAHN6 | | Morin et al. 2010 | | **GU187184** |
| ***Orcinus orca*** | ENAHN3 | | Morin et al. 2010 | | **GU187182** |
| ***Orcinus orca*** | ENAHN2 | | Morin et al. 2010 | | **GU187183** |
| ***Orcinus orca*** | ENAHN8 | | Morin et al. 2010 | | **GU187181** |
| ***Orcinus orca*** | AntA2 | | Morin et al. 2010 | | **GU187155** |
| ***Orcinus orca*** | WNPTRU3 | | Morin et al. 2010 | | **GU187161** |
| ***Orcinus orca*** | ENPTPI2 | | Morin et al. 2010 | | **GU187160** |
| ***Orcinus orca*** | WNPTRU1 | | Morin et al. 2010 | | **GU187159** |
| ***Orcinus orca*** | WNPTRU2 | | Morin et al. 2010 | | **GU187156** |
| ***Orcinus orca*** | WNPTAL2 | | Morin et al. 2010 | | **GU187158** |
| ***Orcinus orca*** | WNPTRU4 | | Morin et al. 2010 | | **GU187157** |
| ***Orcinus orca*** | ENPUCA4 | | Morin et al. 2010 | | **GU187163** |
| ***Orcinus orca*** | ENPTSEA2 | | Morin et al. 2010 | | **GU187162** |
| ***Orcinus orca*** | ENPTAL_GA2 | | Morin et al. 2010 | | **GU187174** |
| ***Orcinus orca*** | CNPUBS | | Morin et al. 2010 | | **GU187170** |
| ***Orcinus orca*** | ENPTAL3 | | Morin et al. 2010 | | **GU187171** |
| ***Orcinus orca*** | ENPTPI1 | | Morin et al. 2010 | | **GU187169** |
| ***Orcinus orca*** | ENPUCA3 | | Morin et al. 2010 | | **GU187167** |
| ***Orcinus orca*** | ENPTCA_ENPUCA2 | | Morin et al. 2010 | | **GU187168** |
| ***Orcinus orca*** | ENPTGA2 | | Morin et al. 2010 | | **GU187174** |
| ***Orcinus orca*** | WNPTAL1_CNPTAL | | Morin et al. 2010 | | **GU187172** |
| ***Orcinus orca*** | ENPTAL2 | | Morin et al. 2010 | | **GU187173** |
| ***Peponocephala electra*** |  | | Vilstrup et al. 2011 | | **JF289175** |
| ***Peponocephala electra*** |  | | Vilstrup et al. 2011 | | **JF289176** |
| ***Phocoena phocoena*** |  | | Arnason et al. 2004 | | **NC005280** |
| ***Physeter catadon*** |  | | Arnason et al. 2004 | | **NC002503** |
| ***Platanista minor*** |  | | Arnason et al. 2004 | | **NC005275** |
| ***Pontoporia blainvillei*** |  | | Arnason et al. 2004 | | **NC005277** |
| ***Pseudorca crassidens*** |  | | Morin et al. 2010 | | **HM060332** |
| ***Pseudorca crassidens*** |  | | Vilstrup et al. 2011 | | **JF289173** |
| ***Pseudorca crassidens*** |  | | Vilstrup et al. 2011 | | **JF289174** |
| ***Sousa chinensis*** |  | | Xiong et al. 2009 | | **EU557091** |
| ***Sousa chiniensis*** |  | | Xiong et al. 2009 | | **EU557P91** |
| ***Stenella attenuata*** |  | | Xiong et al. 2009 | | **EU557096** |
| ***Stenella attenuata*** |  | | Xiong et al. 2009 | | **EU557096** |
| ***Stenella coeruleoalba*** |  | | Xiong et al. 2009 | | **EU557097** |
| ***Stenella coeruleoalba*** |  | | Xiong et al. 2009 | | **EU557097** |
| ***Steno bredanensis*** |  | | Vilstrup et al. 2011 | | **JF339982** |
| ***Tursiops aduncus*** |  | | Xiong et al. 2009 | | **EU55792** |
| ***Tursiops aduncus*** |  | | Xiong et al. 2009 | | **EU557092** |
| ***Tursiops truncatus*** |  | | Xiong et al. 2009 | | **EU557093** |
| ***Tursiops truncatus*** |  | | Xiong et al. 2009 | | **EU557093** |
