# Supplementary material for: Mitogenome Phylogenetics: The Impact of Using Single Regions and Partitioning Schemes on Topology, Substitution Rate and Divergence Time Estimation
Source: PLoS One. 2011 Nov 2;6(11):e27138. doi: 10.1371/journal.pone.0027138 (PMC3206919; doi:10.1371/journal.pone.0027138)
Supplement: Table S2 — Prior distributions used as calibrations for phylogenetic analyses as estimated by Morin et al. 2010 for all sequence Datasets. Values correspond to ages in mya after log transformation. mya = Million years ago. *Calibration priors for Orcinus and Delphinidae sequence datasets correspond to posterior distributions estimated from complete mitogenomic analysis of a Cetacean sequence dataset by Morin et al (2010) and confirmed in this study. Calibrations sequentially removed in “Accuracy of TMRCA estimates for non-calibrated nodes” section for the Cetacea and Delphinidae. (DOC) [file pone.0027138.s004.doc]

**Table S2.** Prior distributions used as calibrations for phylogenetic analyses as estimated by Morin et al. 2010 for all sequence Datasets. Values correspond to ages in mya after log transformation. mya = Million years ago. *Calibration priors for Orcinus and Delphinidae sequence datasets correspond to posterior distributions estimated from complete mitogenomic analysis of a Cetacean sequence dataset by Morin et al (2010) and confirmed in this study. Calibrations sequentially removed in "Accuracy of TMRCA estimates for non-calibrated nodes" section for the Cetacea and Delphinidae.

| **Datasets** | **Taxa** | **Log-normal mean** | **Log-normal s.d.** | **Median (mya)** |
| --- | --- | --- | --- | --- |
| **Cetacea** | Cetacea | 3.67 | 0.07 | 39.20 |
| **Cetacea** | Odontoceti | 3.61 | 0.07 | 36.80 |
| **Cetacea** | Ziphiidae+Delphinida | 3.53 | 0.08 | 34.10 |
| **Cetacea** | Delphinida | 3.37 | 0.08 | 29.10 |
| **Cetacea** | Delphinoidea | 3.10 | 0.10 | 22.20 |
| **Cetacea** | Inioidea | 3.05 | 0.13 | 21.00 |
| **Cetacea** | Phocoenidae+Monodontidae | 2.87 | 0.12 | 17.60 |
| **Cetacea** | Delphinidae | 2.46 | 0.12 | 11.70 |
| **Cetacea** | Delphininae | 1.48 | 0.14 | 4.40 |
| **Cetacea** | *Orcinus* | -0.27 | 0.17 | 0.80 |
| **Delphinidae** | Delphinidae* | 2.56 | 0.07 | 12.90 |
| **Delphinidae** | Delphininae* | 1.63 | 0.09 | 5.20 |
| **Delphinidae + *Orcinus*** | *Orcinus** | -0.35 | 0.17 | 0.70 |
