# Supplementary material for: Mitogenome Phylogenetics: The Impact of Using Single Regions and Partitioning Schemes on Topology, Substitution Rate and Divergence Time Estimation
Source: PLoS One. 2011 Nov 2;6(11):e27138. doi: 10.1371/journal.pone.0027138 (PMC3206919; doi:10.1371/journal.pone.0027138)
Supplement: Table S3 — Substitution models used according to the BIC. (DOCX) [file pone.0027138.s005.docx]

**Table S3.** Substitution models used according to the BIC.

|  | ***Orcinus*** | **Delphinidae** |
| --- | --- | --- |
| ***12S16S*** | HKY | GTR+G |
| ***ND1*** | HKY | HKY+G |
| ***ND2*** | HKY | HKY+G |
| ***COX1*** | HKY | HKY+I+G |
| ***COX2*** | HKY | HKY+G |
| ***ATP8*** | HKY | HKY+G |
| ***ATP6*** | HKY | HKY+G |
| ***COX3*** | HKY | HKY+G |
| ***ND3*** | HKY+G | HKY+G |
| ***ND4L*** | HKY | HKY+G |
| ***ND4*** | HKY | HKY+G |
| ***ND5*** | HKY | HKY+G |
| ***ND6*** | HKY | HKY+G |
| ***CYTB*** | HKY | HKY+G |
| ***CR*** | HKY+G | HKY+I+G |
| ***Complete mitogenome*** | HKY | HKY+G |
