# Supplementary material for: Mitogenome Phylogenetics: The Impact of Using Single Regions and Partitioning Schemes on Topology, Substitution Rate and Divergence Time Estimation
Source: PLoS One. 2011 Nov 2;6(11):e27138. doi: 10.1371/journal.pone.0027138 (PMC3206919; doi:10.1371/journal.pone.0027138)
Supplement: Table S4 — Posterior TMRCA estimates for nodes in Orcinus , Delphinidae and Cetacea datasets using the complete mitogenome. Dates are expressed in Million years before present (MYBP). (DOCX) [file pone.0027138.s006.docx]

**Table S4.** Posterior TMRCA estimates for nodes in *Orcinus*, Delphinidae and Cetacea datasets using the complete mitogenome. Dates are expressed in Million years before present (MYBP).

| **Datasets** | **Taxa** | **Mean** | **Median** | **95% HPD** |
| --- | --- | --- | --- | --- |
| ***Orcinus*** | AntA | 0.16 | 0.14 | 0.02 – 0.36 |
| ***Orcinus*** | AntB | 0.11 | 0.097 | 0.092 – 0.07 |
| ***Orcinus*** | AntC | 0.16 | 0.14 | 0.04 – 0.32 |
| ***Orcinus*** | Offshore | 0.12 | 0.11 | 0.01 – 0.26 |
| ***Orcinus*** | Resident | 0.14 | 0.13 | 0.03 – 0.29 |
| ***Orcinus*** | Atlantic | 0.23 | 0.21 | 0.06 – 0.47 |
| ***Orcinus*** | Transient | 0.23 | 0.20 | 0.06 – 0.45 |
| ***Orcinus*** | *Orcinus* | 0.71 | 0.70 | 0.49 – 0.96 |
| **Delpinidae** | Delphinidae | 12.90 | 12.87 | 11.43 – 14.39 |
| **Delphinidae** | Delphinidae | 5.11 | 5.10 | 4.48 – 5.76 |
| **Delphinidae** | *Globicephala melas* | 0.30 | 0.35 | 0.04 – 0.5 |
| **Delphinidae** | Globicephalinae | 8.08 | 8.07 | 6.95 – 9.25 |
| **Delphinidae** | Globicephala macrorhynchus | 0.34 | 0.33 | 0.24 – 0.44 |
| **Delphinidae** | *Orcinus* | 0.64 | 0.63 | 0.51 – 0.77 |
| **Delphinidae** | *Pseudorca* | 0.22 | 0.22 | 0.13 – 0.30 |
| **Cetacea** | Cetacea | 38.33 | 38.28 | 35.6 – 41.38 |
| **Cetacea** | Delphinida | 27.87 | 27.86 | 25.71 – 30.25 |
| **Cetacea** | Delphinidae | 12.82 | 12.79 | 11.00 – 14.67 |
| **Cetacea** | Delphininae | 5.09 | 5.07 | 4.16 – 5.97 |
| **Cetacea** | Delphinoidea | 21.31 | 21.32 | 19.00 – 23.52 |
| **Cetacea** | Inioidea | 19.29 | 19.32 | 16.47 – 22.14 |
| **Cetacea** | Mysticeti | 23.32 | 23.16 | 18.20 – 28.55 |
| **Cetacea** | Odontoceti | 37.68 | 37.66 | 34.86 – 40.54 |
| **Cetacea** | Phocoenidae + Monodontidae | 17.02 | 17.03 | 14.37 – 19.50 |
| **Cetacea** | *Orcinus* | 0.84 | 0.78 | 0.50 – 1.21 |
| **Cetacea** | Zhiphiidae + Delphinida | 33.20 | 33.15 | 30.50 - |
