# Supplementary material for: Mitogenome Phylogenetics: The Impact of Using Single Regions and Partitioning Schemes on Topology, Substitution Rate and Divergence Time Estimation
Source: PLoS One. 2011 Nov 2;6(11):e27138. doi: 10.1371/journal.pone.0027138 (PMC3206919; doi:10.1371/journal.pone.0027138)
Supplement: Table S5 — Proportion of unique haplotypes detected by each gene for both datasets, alignment length and % similarity. Note that intraspecific sampling coverage is not equivalent between datasets so comparisons of the number of haplotypes for a given gene is not appropriate. (DOCX) [file pone.0027138.s007.docx]

**Table S5:** Proportion of unique haplotypes detected by each gene for both datasets, alignment length and % similarity. Note that intraspecific sampling coverage is not equivalent between datasets so comparisons of the number of haplotypes for a given gene is not appropriate.

|  | ***Orcinus*  Total mitogenomic haplotypes = 64** | **Alignment length** | **% Similarity** | **Delphinidae Total mitogenomic haplotypes = 27** | **Alignment length** | **% Similarity** |
| --- | --- | --- | --- | --- | --- | --- |
| ***12S16S*** | 0.20 | 2550 | 99.8 | 0.78 | 2001 | 94.6 |
| ***ND1*** | 0.28 | 960 | 99.7 | 0.89 | 957 | 94.1 |
| ***ND2*** | 0.20 | 1044 | 99.8 | 0.96 | 1042 | 93.7 |
| ***COX1*** | 0.36 | 1551 | 99.7 | 0.85 | 1351 | 94.7 |
| ***COX2*** | 0.14 | 690 | 99.8 | 0.70 | 684 | 93.9 |
| ***ATP8*** | 0.08 | 207 | 99.2 | 0.70 | 192 | 93.1 |
| ***ATP6*** | 0.22 | 684 | 99.6 | 0.81 | 684 | 92.8 |
| ***COX3*** | 0.19 | 786 | 99.8 | 0.78 | 785 | 94.3 |
| ***ND3*** | 0.14 | 347 | 99.6 | 0.78 | 346 | 92.9 |
| ***ND4L*** | 0.09 | 297 | 99.7 | 0.67 | 297 | 94.3 |
| ***ND4*** | 0.27 | 1378 | 99.7 | 0.93 | 1378 | 93.7 |
| ***ND5*** | 0.30 | 1821 | 99.7 | 0.89 | 1824 | 87.8 |
| ***ND6*** | 0.14 | 524 | 99.4 | 0.19 | 528 | 94 |
| ***CYTB*** | 0.30 | 1140 | 99.7 | 0.85 | 1141 | 87.7 |
| ***CR*** | 0.44 | 1160 | 99.4 | 0.93 | 927 | 84.5 |
| **Mitogenome** | 1 | 16465 | 97.6 | 1 | 16440 | 90.6 |
